# CD4^+^ T cells are activated in regional lymph nodes and migrate to skin to initiate lymphedema

**DOI:** 10.1038/s41467-018-04418-y

**Published:** 2018-05-17

**Authors:** Gabriela D. García Nores, Catherine L. Ly, Daniel A. Cuzzone, Raghu P. Kataru, Geoffrey E. Hespe, Jeremy S. Torrisi, Jung Ju Huang, Jason C. Gardenier, Ira L. Savetsky, Matthew D. Nitti, Jessie Z. Yu, Sonia Rehal, Babak J. Mehrara

**Affiliations:** Division of Plastic and Reconstructive Surgery, Department of Surgery, Memorial Sloan Kettering Cancer Center, 1275 York Avenue, Suite MRI 1006, New York, NY 10065 USA

## Abstract

T cell-mediated responses have been implicated in the development of fibrosis, impaired lymphangiogenesis, and lymphatic dysfunction in secondary lymphedema. Here we show that CD4^+^ T cells are necessary for lymphedema pathogenesis by utilizing adoptive transfer techniques in CD4 knockout mice that have undergone tail skin and lymphatic excision or popliteal lymph node dissection. We also demonstrate that T cell activation following lymphatic injury occurs in regional skin-draining lymph nodes after interaction with antigen-presenting cells such as dendritic cells. CD4^+^ T cell activation is associated with differentiation into a mixed T helper type 1 and 2 phenotype, as well as upregulation of adhesion molecules and chemokines that promote migration to the skin. Most importantly, we find that blocking T cell release from lymph nodes using a sphingosine-1-phosphate receptor modulator prevents lymphedema, suggesting that this approach may have clinical utility.

## Abstract (web summary)

CD4^+^ T cells are critical for the development of lymphedema. Here the authors show how these cells contribute to lymphedema and identify that the sphingosine-1-phosphate receptor modulator FTY720 can prevent lymphedema in a mouse tail injury model by blocking the release of CD4^+^ T cells from the lymph nodes to the skin.

## Introduction

Lymphedema is a morbid disease that commonly occurs after cancer treatment. An estimated 1 in 3 patients who undergo lymphadenectomy for breast cancer will eventually develop the disease^1^. Because lymphedema is associated with poor quality of life and elevated risk of recurrent infections and secondary malignancy, the identification of effective treatment and prevention options is an important clinical goal^2^.

CD4^+^ T cells are known to have a central function in lymphedema. Tekola et al.^3^, for example, highlighted the association between HLA class II loci and podoconiosis, a tropical form of lymphedema, and concluded it may be a T cell-mediated inflammatory disease. Our group has previously shown that CD4^+^ T cell numbers are increased in human lymphedema biopsy samples and, more importantly, that the number of tissue-infiltrating CD4^+^ T cells has a linear positive correlation with disease severity^4^. Using mouse models of lymphedema, we have also demonstrated that, in contrast to wild-type (WT) mice, mice lacking T cells in general (nude mice) or CD4^+^ T cells in particular (CD4 knockout, [CD4KO]) do not develop lymphedema after lymphatic injury^4,5^. Correspondingly, depletion of CD4^+^ T cells, but not CD8^+^ T cells or macrophages, with neutralizing antibodies results in reversal of lymphedema^4,6^. Furthermore, we have found that while lymphedema is characterized by a mixed T helper type 1 (Th1) and T helper type 2 (Th2) infiltrate, Th2 differentiation, specifically, is necessary for pathological changes, such as fibrosis, impaired lymphangiogenesis, and dysfunctional collecting lymphatic vessel pumping and transport function^4,7^. These findings are important and have led to the first human immunotherapy trial analyzing the efficacy of Th2 blockade for the treatment of breast cancer-related lymphedema.

Although it is clear that CD4^+^ T cells are important in lymphedema pathophysiology, few studies have defined the mechanisms regulating T cell activation, differentiation, and homing to lymphedematous tissues. In this study, we show that naive CD4^+^ T cells are activated in skin-draining lymph nodes prior to skin infiltration after interacting with antigen-presenting cells (APC). Activated CD4^+^ T cells then migrate to the skin, where they promote fibrosis and inflammation and negatively regulate lymphangiogenesis and lymphatic function. Consistent with the spatiotemporal patterns of CD4^+^ T cells, we also show that blocking release of T cells from lymph nodes using a sphingosine-1-phosphate receptor modulator is effective at preventing lymphedema in a mouse tail model of lymphatic injury. Our results suggest that this approach may be a promising treatment option for lymphedema, which currently remains without a cure.

## Results

### NK1.1 depletion does not reverse lymphedema

To confirm that CD4^+^ T cells rather than non-conventional T cells are required for lymphedema, we treated WT mice that had undergone tail skin and lymphatic excision with either a monoclonal neutralizing antibody to NK1.1 (a glycoprotein that has a role in natural killer and natural killer T [NKT] cell activation and differentiation^8^) or isotype control (Supplementary Figs. 1, 2a). Mice treated with the antibody starting 2 weeks post-operatively developed tail swelling and fibroadipose deposition similar to that seen in control-treated mice (Supplementary Fig. 2b–e), despite nearly complete absence of NK1.1^+^ cells in the skin (Supplementary Fig. 2f, g). Such data are consistent with previous findings that NKT cells are not significantly increased in mouse models of lymphedema^6^ and indicates that depletion of these cells does not reverse the development of lymphedema.

### CD4^+^ T cells mediate edema after lymphatic injury

**Fig. 1:**
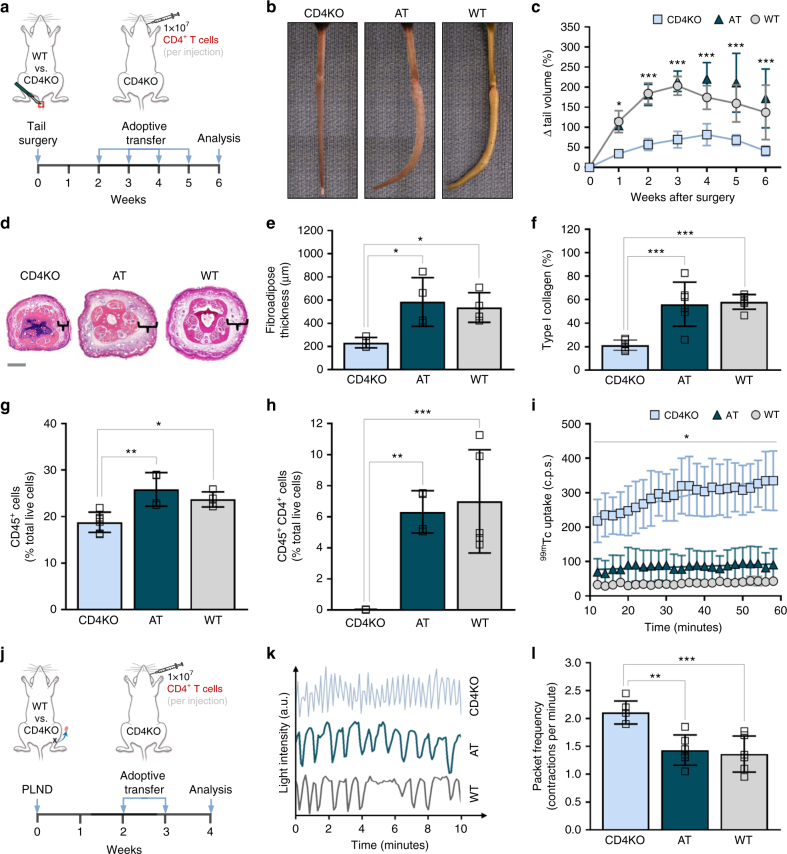
Adoptive transfer of CD4^+^ T cells to CD4KO mice after lymphatic injury results in lymphedema. **a** Schematic diagram of adoptive transfer following tail skin and lymphatic excision. Mice killed 6 weeks after surgery. **b**, **c** Representative photographs of tails (**b**) and quantification of tail volume change (**c**) (*n* = 4 for AT group; *n* = 6 for CD4KO and WT groups; two-way ANOVA with Sidak’s multiple comparisons test). **d** Representative H&E staining of tail cross-sections with brackets indicating fibroadipose tissue; scale bar, 1000 µm. **e** Quantification of fibroadipose thickness (*n* = 4 per group; 4 hpf per mouse). **f** Quantification of tail type I collagen deposition (*n* = 6 per group; 4 hpf per mouse). **g**, **h** FACS quantification of CD45^+^ cells (**g**) and CD45^+^CD4^+^ cells (**h**; *n* = 4 for AT and WT groups, *n* = 6 for CD4KO group; 4 hpf per mouse). **i** Quantification of decay-adjusted ^99m^Tc uptake by sacral lymph nodes after distal tail injection (*n* = 4 per group; mean ± s.e.m.; two-way ANOVA with Sidak’s multiple comparisons test). **j** Schematic diagram of adoptive transfer following PLND. Mice harvested 4 weeks after surgery. **k** Representative graphs collecting lymphatic vessel pumping over time as determined by changes in ICG light intensity. **l** Quantification of collecting lymphatic vessel contractions per minute (*n* = 6 per group). Data representative of a minimum of two independent experiments with similar results; statistical analyses of one experiment shown. Mean ± s.d.; *P < 0.05, **P < 0.01, and ***P < 0.001 by one-way ANOVA with Tukey’s multiple comparisons test, unless otherwise specified. AT, CD4KO mice that underwent adoptive transfer with naive CD4^+^ T cells; a.u. arbitrary units; c.p.s, counts per second; FACS, fluorescence-activated cell sorting; H&E, hematoxylin and eosin; hpf, high-powered field; ^99m^Tc, technetium-99m sulfur colloid; PLND, popliteal lymph node dissection; ICG, indocyanine green

Knowing that the absence of CD4^+^ T cells prevents lymphedema^4,9^, we then evaluated if adoptive transfer of naive CD4^+^ T cells from WT mice (Supplementary Figs. 1, 3) to CD4KO mice after lymphatic injury was sufficient to induce lymphedema. We studied this using both the tail surgery model of lymphedema (Fig. 1a), which is useful for evaluation of histological features such as edema, fibrosis, and inflammation, and the popliteal lymph node dissection (PLND) model (Fig. 1j), which is valuable for analysis of physiologic changes such as lymphangiogenesis and lymphatic function^10^.

CD4KO mice that underwent adoptive transfer (henceforth referred to as AT mice) after tail skin and lymphatic excision developed tail edema nearly identical to that in similarly injured WT mice (Fig. 1b, c). In contrast, CD4KO mice that endured lymphatic injury without subsequent adoptive transfer had minimal tail edema that was significantly less than that noted in the AT and WT mice (Fig. 1b, c).

### CD4^+^ T cells mediate fibrosis after lymphatic injury

Both AT and WT mice also had similar increases in tail fibroadipose thickness compared to CD4KO mice (Fig. 1d, e). This correlated with extensive accumulation of type I collagen (Fig. 1f; Supplementary Fig. 4a**)**. Looking more closely, we found that collagen fibers virtually encased the dermal lymphatic capillary vessels. In contrast, the tails of CD4KO mice demonstrated sparse staining for type I collagen overall and virtually no perilymphatic collagen fibers after surgery.

Fibrosis in lymphedema is associated with progressive accumulation of alpha-smooth muscle actin (α-SMA) around podoplanin-expressing collecting lymphatic vessels, which are eventually obliterated in late stages of the disease^11^. Such vessels are easily identified in mouse hindlimbs, so we assessed α-SMA accumulation in CD4KO, AT, and WT mice after PLND. Consistent with our collagen results, the collecting lymphatic vessels in AT and WT mice had a more collapsed appearance compared to those in CD4KO animals (Supplementary Fig. 4b). In addition, both AT and WT mice had more than a twofold increase in perilymphatic α-SMA thickness (Supplementary Fig. 4c).

### CD4^+^ T cells mediate inflammation after lymphatic injury

Analysis of the inflammatory cell infiltrate in the tail skin of AT mice that had undergone tail skin and lymphatic excision revealed that these mice also developed inflammation comparable to that in WT mice. Compared to CD4KO mice, AT mice had a greater number of CD45^+^ leukocytes in general (Fig. 1g; Supplementary Fig. 4d) and CD4^+^ cells specifically (Fig. 1h; Supplementary Fig. 4e).

### CD4^+^ T cells impair lymphangiogenesis after lymphatic injury

Because T cell-derived cytokines such as interferon gamma (IFN-γ), interleukin-4 (IL-4), IL-13, and transforming growth factor-beta1 (TGF-β1) have potent anti-lymphangiogenic effects^7^, we suspected that CD4KO mice would also have significantly more patent lymphatic vessels compared to AT and WT mice. Consistent with this hypothesis, indocyanine green (ICG) lymphangiography of the hindlimbs of CD4KO mice that had undergone PLND revealed extensive collateral lymphatic vessel formation with drainage towards the inguinal lymph nodes, while AT and WT mice had relatively few vessels (Supplementary Fig. 5a). These observations were confirmed with histological sections of hindlimb skin showing a nearly twofold increase in LYVE-1^+^ lymphatic vessels in CD4KO mice, as compared with AT and WT mice (Supplementary Fig. 5b, c).

### CD4^+^ T cells impair lymphatic function after lymphatic injury

In lymphedema, the lymphatic vasculature is not only abnormal because its structure, but also because it is dysfunctional^12^. Therefore, it was not surprising that injection of technetium-99m sulfur colloid (^99m^Tc) into the distal tails of AT and WT mice after tail skin and lymphatic excision resulted in decreased transport to the sacral lymph nodes compared to their CD4KO counterparts (Fig. 1i). Similarly, when we used ICG lymphangiography to quantify the number of hindlimb collecting lymphatic vessel contractions per minute (packet frequency) in PLND-operated mice, we found that, compared to CD4KO mice, AT and WT mice had notably diminished lymphatic pumping (Fig. 1k, l; Supplementary Movies 1–3). Taken together, these findings suggest that CD4^+^ T cells promote lymphatic dysfunction after lymphatic injury, at least in part by increasing fibrosis and perilymphatic inflammation and inhibiting formation of functional collateral lymphatics.

### CD4^+^ T cells differentiate into a mixed Th1/Th2 phenotype

**Fig. 2:**
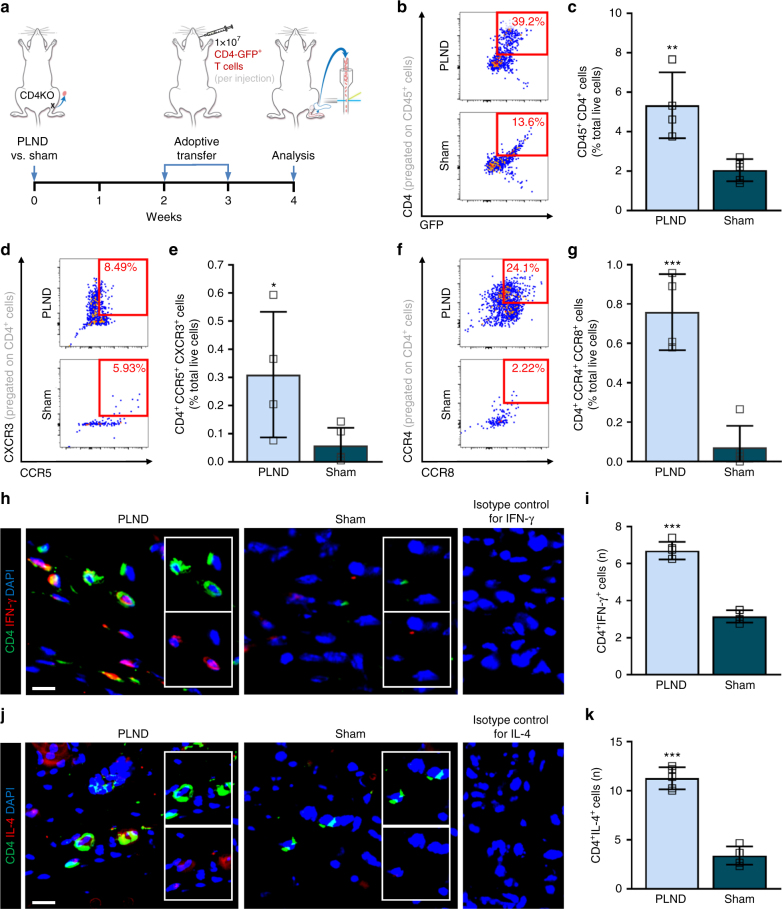
Adoptively transferred CD4^+^ T cells differentiate into a mixed Th1/Th2 phenotype. **a** Schematic diagram of adoptive transfer following PLND versus sham surgery. Mice killed 4 weeks after surgery. **b**, **c** Representative FACS plots (**b**) and quantification (**c**) of single, live CD45^+^CD4^+^ cells (*n* = 4 for PLND, *n* = 5 for sham). **d**, **e** Representative FACS plots (**d**) and quantification (**e**) of single, live CD4^+^CCR5^+^CXCR3^+^ Th1 cells (*n* = 4 for PLND, *n* = 5 for sham). **f**, **g** Representative FACS plots (**f**) and quantification (**g**) of single, live CD4^+^CCR4^+^CCR8^+^ Th2 cells (*n* = 4 for PLND, *n* = 5 for sham). **h** Representative immunofluorescent images co-localizing CD4 and IFN-γ with inset images demonstrating CD4 alone (inset upper) and IFN-γ alone (inset lower); scale bar, 10 µm. Isotype control refers to use of control for IFN-γ in mice without CD4-GFP expression. **i** Quantification of CD4^+^IFN-γ^+^ cells per 0.25 mm^2^ (*n* = 5 per group; 4–5 hpf per mouse). **j** Representative immunofluorescent images co-localizing CD4 and IL-4 with inset images demonstrating CD4 alone (inset upper) and IL-4 alone (inset lower); scale bar, 10 µm. Isotype control refers to use of control for IL-4 in mice without CD4-GFP expression. **k** Quantification of CD4^+^IL-4^+^ cells per 0.25 mm^2^ (*n* = 5 per group; 4–5 hpf per mouse). Data representative of a minimum of two independent experiments with similar results; statistical analyses of one experiment shown. Mean ± s.d.; *P < 0.05, **P < 0.01, and ***P < 0.001 by unpaired student’s *t*-test. FACS, fluorescence-activated cell sorting; hpf, high-powered field; PLND, popliteal lymph node dissection; Th1, T helper type 1; Th2, T helper type 2

Studies have shown that lymphedema is associated with cutaneous infiltration of both Th1 and Th2 cells^4,6^. To determine whether adoptively transferred naive CD4^+^ T cells follow a similar differentiation pattern, we transferred naive CD4-GFP^+^ T cells to CD4KO mice after PLND or sham surgery (incision in the popliteal area without lymphadenectomy) (Fig. 2a). Consistent with our previous results, we found that PLND-treated mice had significantly more CD45^+^CD4^+^ T cells in the ipsilateral hindlimb skin compared to their sham-operated counterparts (Fig. 2b, c; Supplementary Fig. 1). Further flow cytometric characterization of these cells revealed a 6.7-fold increase in CD4^+^CXCR3^+^CCR5^+^ Th1 cells and a 10.5-fold increase in CD4^+^CCR4^+^CCR8^+^ Th2 cells in PLND-operated mice (Fig. 2d, e; Supplementary Fig. 1). These findings were corroborated by immunohistochemical analysis of hindlimb tissues demonstrating significantly more CD4^+^IFN-γ^+^ cells (putative Th1 cells) (Fig. 2h, i) and CD4^+^IL-4^+^ cells (putative Th2 cells) in animals that underwent PLND (Fig. 2j, k; Supplementary Fig. 1).

### Systemic CD4^+^ T cells migrate from lymph nodes to skin

**Fig. 3:**
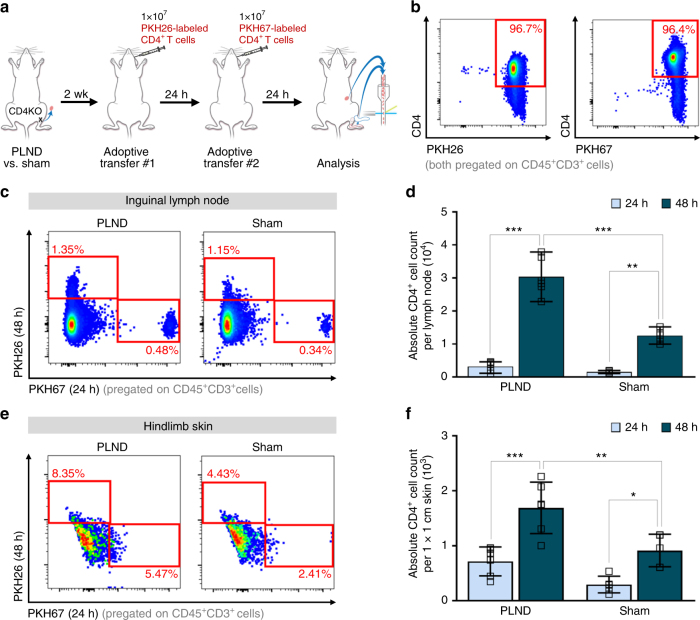
Adoptively transferred CD4^+^ T cells migrate to skin from regional lymph nodes. **a** Schematic diagram of adoptive transfer following PLND versus sham surgery. Mice killed 16 days after surgery. **b** Representative FACS plots of single, live CD45^+^CD3^+^CD4^+^ cells labeled with either PKH26 or PKH67 after naive CD4^+^ T cell isolation from the spleens of WT mice. **c**, **d** Representative FACS plots (**c**) and quantification (**d**) of single, live CD45^+^CD3^+^PKH67^+^ cells (injected 24 h prior) and CD45^+^CD3^+^PKH26^+^ cells (injected 48 h prior) in ipsilateral inguinal lymph nodes (*n* = 6 for PLND, *n* = 5 for sham). **e**, **f** Representative FACS plots (**c**) and quantification (**d**) of single, live CD45^+^CD3^+^PKH67^+^ cells (injected 24 h prior) and CD45^+^CD3^+^PKH26^+^ cells (injected 48 h prior) in ipsilateral distal hindlimb skin (*n* = 6 for PLND, *n* = 5 for sham). Data representative of a minimum of two independent experiments with similar results; statistical analyses of one experiment shown. Mean ± s.d.; *P < 0.05, **P < 0.01, and ***P < 0.001 by one-way ANOVA with Tukey’s multiple comparisons test. AT, CD4KO mice that underwent adoptive transfer with naive CD4^+^ T cells; FACS, fluorescence-activated cell sorting; PLND, popliteal lymph node dissection

It is well-known that lymphocytes continually circulate and re-enter peripheral lymphoid organs until they either encounter antigen or die^13^. Antigen-experienced effector CD4^+^ T cells then home to target tissues through a series of interactions with proteins that guide migration^14^. On the basis of this knowledge, we hypothesized that adoptive transfer of naive CD4^+^ T cells after lymphatic injury would result in initial accumulation of CD4^+^ T cells in the lymph nodes followed by migration to the skin. To study this, we compared CD4^+^ T cell populations in the ipsilateral inguinal lymph nodes (immediately upstream of the popliteal lymph nodes^15^) and hindlimb skin distal to the surgical site 24 and 48 h after adoptive transfer of naive CD4^+^ T cells to PLND- or sham-operated CD4KO mice (Fig. 3a). Isolated CD4^+^ T cells were fluorescently labeled using cell-linkage technology to allow for tracking of cells injected at different times (Fig. 3b).

First looking at the 24-h timepoint, we found that there was fourfold increase in CD4^+^ T cells in the lymph nodes compared to the skin (Fig. 3c–f; Supplementary Fig. 1). In addition, we noted there were greater than two times more CD4^+^ T cells in the lymph nodes and skin of PLND-treated mice compared to sham-operated controls, but these differences were not statistically significant (Fig. 3c–f; Supplementary Fig. 1). Importantly, these relationships were all markedly exaggerated by 48 h, at which time the number of CD4^+^ T cells had increased in both the lymph nodes and skin, particularly in PLND-treated mice. This is likely reflective of the time required for the accumulation of cells after adoptive transfer. At the 48-h timepoint, there were significantly more CD4^+^ T cells in both the lymph nodes and skin of PLND-treated mice compared to sham-operated controls (Fig. 3c–f; Supplementary Fig. 1). Furthermore, there were nearly 20 times the number of CD4^+^ T cells in the lymph node than the skin (Fig. 3c–f; Supplementary Fig. 1). Taken together, such spatiotemporal sequences suggest that CD4^+^ T cells are activated in the lymph nodes, are released by the lymph nodes, and then migrate to the skin, resulting in an increase in skin CD4^+^ T cells over time.

### Lymphatic injury promotes migration of CD4^+^ T cells to skin

**Fig. 4:**
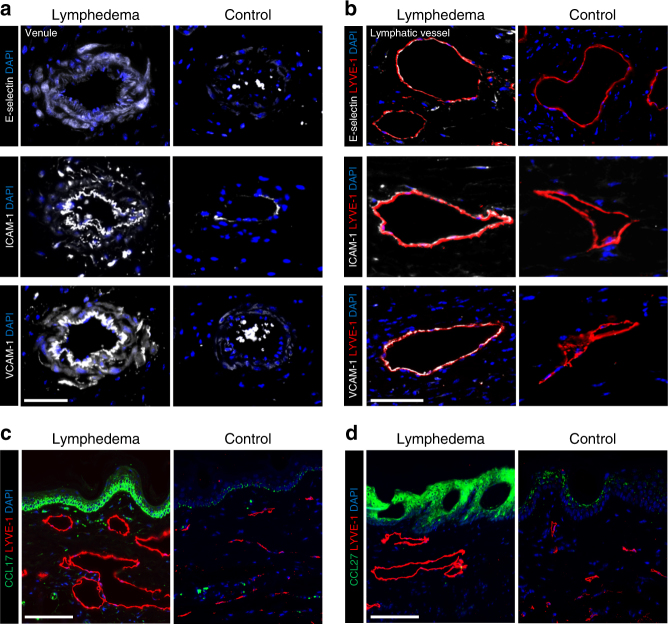
Lymphatic injury results in upregulation of adhesion molecules and chemokines in lymphedematous skin. Mice killed 6 weeks after tail skin and lymphatic excision (lymphedema) or sham surgery (control). **a**, **b** Representative immunofluorescent images of venules (**a**) and LYVE-1^+^ lymphatic vessels (**b**) in tail cross-sections with localization of E-selectin (top), ICAM-1 (middle), and VCAM-1 (bottom); scale bars, 50 and 30 µm, respectively. **c**, **d** Representative immunofluorescent images of tail cross-sections co-localizing LYVE-1^+^ lymphatic vessels with CCL17 (**c**) and CCL27 (**d**); scale bars, 100 µm

Leukocyte adhesion molecules such as E-selectin, ICAM-1, and VCAM-1 bind CD4^+^ T cell receptors such as cutaneous leukocyte antigen (CLA) and CD11a to arrest them in local blood and lymphatic vessels, thus allowing for transmigration into tissues^16^. Similarly, homing of inflammatory cells to the skin is regulated by upregulation of CCL17, which binds CCR4, in the epidermis, dermis, and skin capillaries^17^, as well as increased keratinocyte expression of CCL27, which binds CCR10 on CD4^+^ T cells^18^. To determine whether these adhesion molecules and chemokines are increased in lymphedema, we analyzed tails 6 weeks after skin and lymphatic excision and compared them with controls collected from mice treated only with skin incision. We chose tail specimens for these experiments because the degree of lymphedema in this model is more severe than that in the PLND model. Analysis of both venules (Fig. 4a) and LYVE-1^+^ capillary lymphatic vessels (Fig. 4b) revealed marked increases in E-selectin, VCAM-1, and ICAM-1 in animals that endured lymphatic injury as compared to controls. In addition, compared to controls, CCL17 and CCL27 expression were notably elevated in lymphedematous tail skin keratinocytes (Fig. 4c, d). CCL17 was also highly expressed dermal cells closely associated with capillary lymphatics. Collectively, these findings suggest that lymphatic injury results in upregulation of leukocyte adhesion molecules and chemokines to guide CD4^+^ T cell migration to lymphedematous skin.

### Systemic DCs are initially activated in the skin

**Fig. 5:**
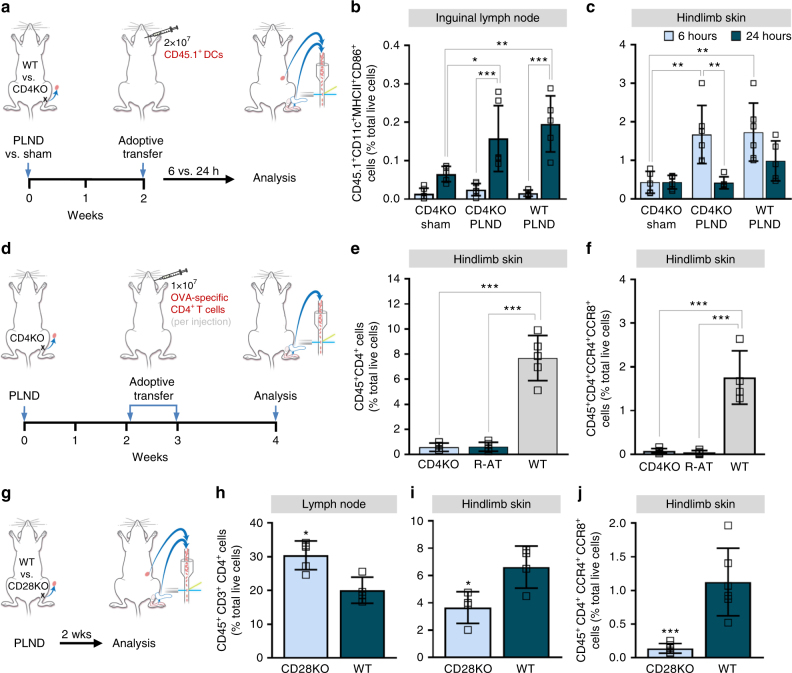
CD4^+^ T cell activation is required for lymphedema. **a** Schematic diagram of adoptive transfer of CD45.1^+^ DCs following PLND or sham surgery. Mice killed 2 weeks after surgery. **b**, **c** FACS quantification of CD45.1^+^CD11c^+^MHCII^+^CD86^+^ cells in ipsilateral inguinal lymph nodes (**b**) and hindlimb skin after PLND or sham surgery with adoptive transfer of CD45.1^+^ DCs (**c**; *n* = 6 per group). **d** Schematic diagram of adoptive transfer of ovalbumin-specific CD4^+^ T cells following PLND. Mice killed 4 weeks after surgery. **e**, **f** FACS quantification of CD45^+^CD4^+^ cells (**e**) and CD45^+^CD4^+^CCR4^+^CCR8^+^ Th2 cells (**f**) in ipsilateral hindlimb skin after PLND with and without adoptive transfer with ovalbumin-specific CD4^+^ T cells (*n* = 5 per group). **g** Schematic diagram of PLND in WT or CD28KO mice. Mice killed 2 weeks after surgery. **h**, **i** FACS quantification of CD45^+^CD3^+^CD4^+^ cells in ipsilateral inguinal lymph nodes (**h**) and hindlimb skin after PLND (**i**; *n* = 5 per group). **j** FACS quantification of CD45^+^CD4^+^CCR4^+^CCR8^+^ Th2 cells in ipsilateral hindlimb skin after PLND (*n* = 6 per group). Data representative of a minimum of two independent experiments with similar results; statistical analyses of one experiment shown. Mean ± s.d.; *P < 0.05, **P < 0.01, and ***P < 0.001 by one-way ANOVA with Tukey’s multiple comparisons tests or unpaired student’s *t*-test. DCs, dendritic cells; FACS, fluorescence-activated cell sorting; PLND, popliteal lymph node dissection

APCs such as dendritic cells (DC) have a central function in the activation of tissue-trophic effector T cell subsets^19^. To examine the spatiotemporal sequence of systemic DCs in lymphedema, we performed adoptive transfer of CD45.1^+^CD11c^+^ cells to CD45.2^+^ CD4KO and WT mice that had undergone PLND or sham surgery and compared the activation status of these cells (as indicated by CD86 expression) in the ipsilateral inguinal lymph nodes and hindlimb skin 6 and 24 h later (Fig. 5a). We found that there were no significant differences in the proportion of CD45.1^+^CD11c^+^MHCII^+^CD86^+^ DCs in the lymph nodes between the groups until 24 h after adoptive transfer. At 24 h post-transfer, the number of CD45.1^+^CD11c^+^MHCII^+^CD86^+^ DCs in the lymph nodes had increased significantly in both the CD4KO and WT mice that had undergone PLND, but not the CD4KO mice that endured only sham surgery (Fig. 5b; Supplementary Fig. 6a). In contrast, there were significantly more activated DCs in the skin of PLND-operated CD4KO and WT mice compared to sham-operated mice by 6 h after adoptive transfer (Fig. 5c; Supplementary Fig. 6b) with a subsequent decrease over time. These results suggest that lymphatic injury promotes the migration of systemic DCs to the skin in the area of injury, where they become activated, with subsequent accumulation in regional lymph nodes.

### CD4^+^ T cell activation is required for lymphedema

With evidence demonstrating that systemic DCs travel from the skin to the lymph nodes with subsequent migration of CD4^+^ T cells from the lymph nodes to the skin, we then hypothesized that T cell receptor (TCR) activation is critical to the development of lymphedema. To confirm this, we conducted adoptive transfer of CD4^+^ cells of fixed specificity. Instead of WT mouse-derived naive CD4^+^ T cells, we transferred ovalbumin-specific naive CD4^+^ T cells from transgenic RAG2/OTII mice to CD4KO mice after PLND (henceforth referred to as R-AT mice) (Fig. 5d). CD4KO mice that did not undergo adoptive transfer and WT mice were used as controls. In contrast to WT mice, R-AT mice had significantly fewer CD45^+^CD4^+^ T cells in the hindlimb skin (Fig. 5e; Supplementary Fig. 1, 7a); in fact, R-AT mice appeared similar to CD4KO mice. More specifically, R-AT mice had fewer CD45^+^CD4^+^CCR4^+^CCR8^+^ Th2 cells in the hindlimb skin compared to WT mice (Fig. 5f; Supplementary Fig. 1, 7b). These findings indicate that T cell receptor activation is required to initiate lymphedema pathology. Furthermore, this suggests that these T cells are responding to a specific antigen that is yet to be determined.

Full activation of T cells cannot occur without interaction between the CD28 molecule, which is constitutively expressed on CD4^+^ T cells, and its B7 ligand (CD86 on DCs)^20^. On the basis of this knowledge, we then hypothesized that the absence of CD28 would prevent lymphedema, thus further confirming that TCR activation is necessary for disease development. To study this, we performed PLND in CD28 knockout (CD28KO) and WT mice (Fig. 5g). In support of our hypothesis, we found that CD28KO mice had significantly fewer CD4^+^ T cells in general (Fig. 5i; Supplementary Fig. 7d) and CD4^+^CCR4^+^CCR8^+^ Th2 cells in particular (Fig. 5j; Supplementary Fig. 7e) in the hindlimb skin as compared to WT mice. Interestingly, the CD28KO mice had more CD4^+^ T cells in the ipsilateral inguinal lymph node as compared to WT mice, possibly reflecting the presence of naive T cells that cannot be activated due to the absence of CD28 (Fig. 5h; Supplementary Fig. 7c). Taken together, our findings suggest that circulating DCs rapidly migrate to lymphedematous tissues, become activated, and then traffic to regional lymph nodes where two-signal interaction with T cells is necessary for T cell differentiation.

### CD4^+^ T cells inhibit lymphatic pumping via increased iNOS

**Fig. 6:**
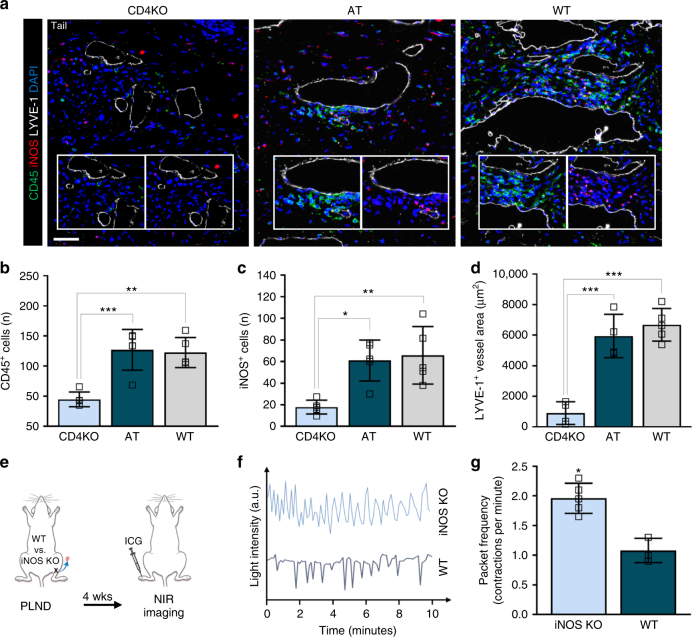
Adoptive transferred CD4^+^ T cells mediate iNOS production by macrophages after lymphatic injury. **a** Mice killed 6 weeks after tail skin and lymphatic excision and 1 week after the last adoptive transfer of CD4^+^ T cells for AT mice. Representative immunofluorescent images of tail cross-sections co-localizing LYVE-1^+^ initial lymphatic vessels with CD45 and iNOS; inset images demonstrate CD45 alone (inset left) and iNOS alone (inset right); scale bar, 50 µm. **b**, **c** Quantification of CD45^+^ (**b**) and iNOS^+^ cells (**c**) within 50 µm of LYVE-1^+^ lymphatic vessels (*n* = 5 per group; 4 hpf per mouse). **d** Quantification of LYVE-1^+^ lymphatic vessel area (*n* = 4 for CD4KO and AT groups, *n* = 5 for WT group; 4 hpf per mouse). **e** Schematic diagram of PLND in WT versus iNOS KO mice. Mice analyzed by NIR imaging 4 weeks after surgery. **f** Representative graphs of collecting lymphatic vessel pumping as determined by changes in ICG light intensity. **g** Quantification of collecting lymphatic vessel contractions per minute (*n* = 5 per group). Data representative of a minimum of two independent experiments with similar results; statistical analyses of one experiment shown. Mean ± s.d.; *P < 0.05, **P < 0.01, and ***P < 0.001 by one-way ANOVA with Tukey’s multiple comparisons test. AT, CD4KO mice that underwent adoptive transfer with naive CD4^+^ T cells; hpf, high-powered field; NIR, near-infrared; PLND, popliteal lymph node dissection

We next aimed to elucidate the mechanisms by which CD4^+^ T cells promote lymphedema. Evidence has shown that inflammatory stimuli decrease lymphatic contractility, at least in part, by increased expression of induced nitric oxide synthase (iNOS) by inflammatory cells with resultant disruption of endogenous nitric oxide (NO) gradients ordinarily regulated by endothelial-derived nitric oxide synthase (eNOS)^21,22^. Consistent with this, distal tail tissues demonstrated a marked increase in perilymphatic accumulation of inflammatory cells in AT and WT mice compared to CD4KO mice with a corresponding increase in iNOS^+^ cells (Fig. 6a–c). We also noted that the lymphatic vessels in AT and WT mice were significantly more dilated than that in CD4KO mice (Fig. 6d).

Knowing that lymphedema is associated with iNOS accumulation and that increased iNOS affects lymphatic pumping, we suspected that the absence of iNOS would prevent impairment of lymphatic pumping after injury. We assessed this by evaluating the effect of PLND on WT mice compared to iNOS knockout (iNOS KO) mice (Fig. 6e). We found that, 4 weeks after surgery, iNOS KO mice had a 1.8-fold increase in packet frequency as compared to WT mice (Fig. 6f, g; Supplementary Movie 4). In fact, iNOS KO mice appeared to have a similar packet frequency to that observed in CD4KO mice (compare with Fig. 1j).

Because macrophages are well-characterized producers of iNOS, we also evaluated the presence of these particular cells in lymphedema. Consistent with our prior data, both AT and WT mice had significantly more perilymphatic CD11b^+^ macrophages as compared to CD4KO mice (Supplementary Fig. 8a, b). Then, to test our hypothesis that T cell-derived cytokines promote the accumulation of iNOS-producing macrophages, we treated bone marrow-derived monocytes from WT mice with IFN-γ (produced by Th1 cells) alone, IL-4 (produced by Th2 cells) alone, a combination of IFN-γ and IL-4, or macrophage colony-stimulating factor (M-CSF) alone without cytokines as a control (Supplementary Fig. 8c, d). Using polymerase chain reaction (PCR) analysis, we found that in vitro treatment of monocytes with IFN-γ and IL-4 combined resulted in significant upregulation of iNOS and Arginase-1 (Supplementary Fig. 8e), which are indicative of M1 and M2 macrophages, respectively^23^. This was corroborated by immunofluorescent staining demonstrating increased iNOS in the combined treatment group compared to control (Supplementary Fig. 8f); of note, flattened expanded iNOS^−^ cells consistent with the phenotypic appearance of M2 cells^24^ were also noted in the combined group but are not shown. This mixed macrophage population parallels our prior findings of the mixed Th1 and Th2 phenotype found in lymphedema (Fig. 2) and is supported by reports demonstrating the delicate balance between Th1/Th2 cells and M1/M2 cells^25^.

### Blocking lymph node CD4^+^ T cell release prevents lymphedema

**Fig. 7:**
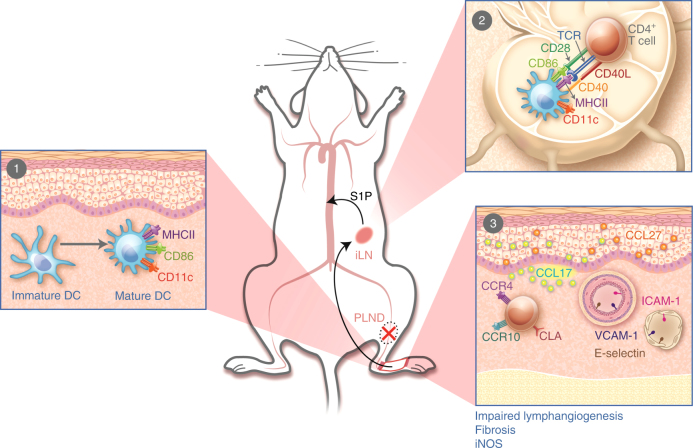
Proposed pathophysiology of secondary lymphedema. **1** After PLND, DCs activate in the area of lymphatic injury with subsequent migration to the nearest skin-draining lymph node(s). **2** DCs interact with and activate CD4^+^ T cells in the skin-draining lymph node(s). Activated CD4^+^ T cells are then released from the lymph node(s) into the systemic circulation. **3** Activated CD4^+^ T cells preferentially migrate to the skin in the area of lymphatic injury by following gradients created by upregulation of leukocyte adhesion molecules on blood and lymphatic vessels (e.g., E-selectin, ICAM-1, and VCAM-1) and chemokines like CCL17 and CCL27, all of which bind skin-homing receptors such as CCR4, CCR10, and CLA. Once in the skin, CD4^+^ T cells promote the development of features such as impaired lymphangiogenesis, fibrosis, and increased iNOS expression. ILN, inguinal lymph node; PLND, popliteal lymph node dissection; S1P, sphingosine-1-phosphate

**Fig. 8:**
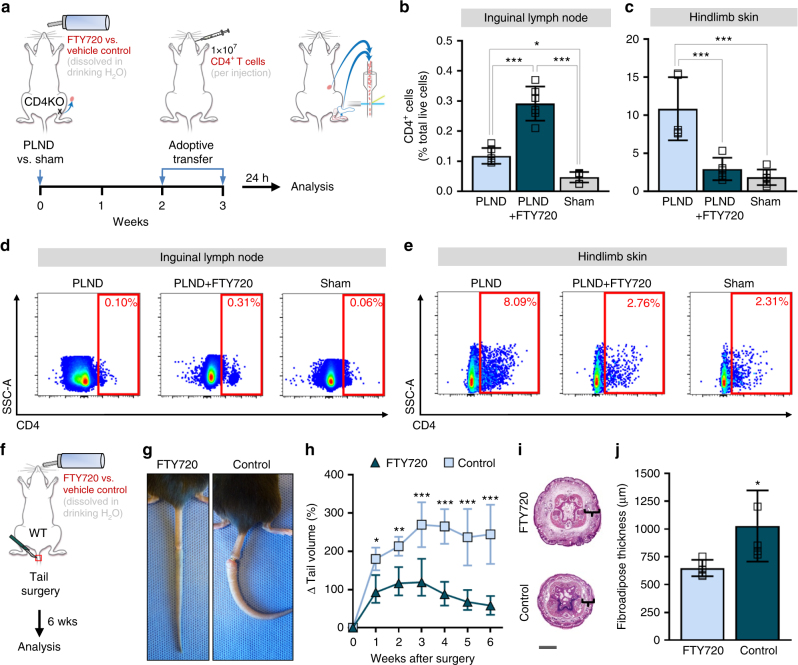
Inhibition of CD4^+^ T cell release from lymph nodes prevents lymphedema. **a** Schematic diagram of adoptive transfer following PLND or sham surgery in the setting of FTY720 treatment. Mice killed 3 weeks after surgery. **b**, **c** FACS quantification of CD4^+^ cells in ipsilateral inguinal lymph nodes (**b**) and hindlimb skin (**c**) (n = 6 per group). **d**, **e** Representative FACS plots of single, live CD4^+^ cells in ipsilateral inguinal lymph nodes (**d**) and hindlimb skin (**e**). **f** Schematic diagram of FTY720 treatment in WT mice following tail surgery. Mice killed 6 weeks after surgery. **g** Representative photographs of tails. **h** Quantification of tail volume change (*n* = 5 per group). **i** Representative H&E staining of tail cross-sections with brackets indicating fibroadipose tissue; scale bar, 2000 µm. **j** Quantification of fibroadipose thickness (*n* = 5 per group; 4 hpf per mouse). Data representative of a minimum of two independent experiments with similar results; statistical analyses of one experiment shown. Mean ± s.d.; *P < 0.05, **P < 0.01, and ***P < 0.001 by one-way ANOVA with Tukey’s multiple comparisons test or unpaired student’s *t-*test.  FACS, fluorescence-activated cell sorting; H&E, hematoxylin and eosin; PLND, popliteal lymph node dissection

 Given our findings of the spatiotemporal patterns of CD4^+^ T cells in lymphedema (Fig. 7), we hypothesized that inhibition of the release of CD4^+^ T cells from lymph nodes would prevent lymphedema. To test this, we treated PLND-operated AT mice with FTY720, a sphingosine-1-phosphate (S1P) receptor modulator known to sequester lymphocytes in lymph nodes^26^, or vehicle control (Fig. 8a). Untreated sham-operated mice were used as controls. Consistent with the known mechanism of FTY720, we found that FTY720-treated PLND-operated mice had significantly more CD4^+^ T cells in the ipsilateral inguinal lymph nodes compared to those treated with control (Fig. 8b, d). More importantly, FTY720 treatment resulted in significantly fewer CD4^+^ T cells in the hindlimb skin after PLND (Fig. 8c, e); in fact, FTY720-treated PLND-operated mice had a similar proportion of skin CD4^+^ T cells as untreated sham-operated mice.

To determine whether FTY720 treatment can be useful for treating lymphedema, we tested the effect of FTY720 treatment on lymphedema development after tail skin and lymphatic excision (Fig. 8f). After 6 weeks of treatment, FTY720-treated mice had markedly decreased tail edema (Fig. 8g, h) and fibroadipose deposition (Fig. 8i, j) compared to control-treated mice, which developed the expected lymphedema phenotype. Taken together, these findings confirm that the migration of CD4^+^ T cells from lymph nodes to skin is critical to lymphedema. Importantly, such data demonstrate that sequestration of immune cells in lymph nodes using a treatment such as FTY720 prevents the migration of these pathologic cells to the skin and therefore hinders disease development.

## Discussion

CD4^+^ T cells comprise a significant portion of the inflammatory infiltrate in lymphedematous skin^4,6,27^. In this study, we confirm that non-conventional T cells such as NKT cells do not have as great of a role as traditional CD4^+^ T cells by demonstrating that NK1.1 depletion does not reverse lymphedema in the same way that CD4 depletion does. It is possible that NK1.1^+^ cells contribute in a different manner, but we also provide evidence that CD4^+^ T cells mediate fibroadipose deposition, impaired collateral vessel formation, dysfunctional lymphatic transport, and diminished lymphatic vessel pumping. Furthermore, we show that these important cells require activation in nearby draining lymph nodes by APCs with resultant differentiation into a mixed Th1/Th2 phenotype and upregulation of adhesion molecules and chemokines that guide them specifically to the skin distal to the zone of lymphatic injury. Collectively, our results suggest that lymphatic injury results in tissue changes that drive CD4^+^ T cell activation and differentiation in skin-draining lymph nodes and that this immunologic response has a key role in lymphedema pathogenesis (Fig. 7).

The adoptive transfer experiments enabled us to investigate the spatiotemporal patterns of CD4^+^ T cell activation after lymphatic injury. By analyzing the presence of CD4^+^ T cells in ipsilateral inguinal lymph node and the skin distal to the surgical site at different time points in CD4KO mice that underwent adoptive transfer following PLND, we found that systemic naive CD4^+^ T cells first migrate to lymph nodes spatially close to the zone of lymphatic injury, become activated, and then are released into the systemic circulation. It is important to note that while the PLND model of lymphedema does not result in significant swelling, in contrast to the tail surgery model, it is more like the clinical scenario of lymph node dissection and results in a similar inflammatory response as noted in the tail surgery model and clinical specimens.

Naive CD4^+^ T cells preferentially migrate through secondary lymphoid organs, where they encounter their cognate antigens and interact with APCs. Although a minority also migrates through peripheral tissues, the functional consequence of this migration is debated^28^. The activation of naive CD4^+^ T cells is the consequence of a complex process initiated by the interaction between CD4 and TCR on lymphocytes with the antigen–MHCII complex on APCs^29^. DCs are the most important and efficient APCs for the activation of naive CD4^+^ T cells, but macrophages and B cells also contribute to this process^30^. In this study, we specifically tracked systemic DCs using a modified adoptive transfer protocol (Fig. 5a) and found that early accumulation of activated donor DCs in the skin decreased over time and correlated with an increase in inguinal lymph nodes, thus suggesting initial activation in the skin with later migration to the lymph nodes (Fig. 5b, c). It is likely that tissue-resident DCs (which are CD45.2^+^) have a role in T cell activation, but our data suggest that systemic DCs migrate to lymphedematous tissues and also participate in this process.

The molecular basis of the interaction between APCs and naive CD4^+^ T cells is not completely understood, but is known that the optimal immune response requires CD28 co-stimulation in addition to TCR stimulation^20^. Knowing this, we confirmed our hypothesis that CD4^+^ T cells must first be activated to promote lymphedema by demonstrating that the absence of the appropriate cognate antigen for the TCR or CD28 prevents the accumulation of the distinct inflammatory infiltrate seen in lymphedematous skin (Fig. 5g–j). Of note, we attempted to test the effect of MHCII blockade on lymphedema, but, like others, found that we were unable to inhibit MHCII to a sufficient degree^31^. Regardless, our findings that activated CD4^+^ T cells are necessary suggest they may be reactive to specific antigens. Further research is necessary to identify these antigens.

Once activated, some T cells remain in lymph nodes to activate B cell responses, while others exit via efferent vessels and infiltrate peripheral tissues by expression of homing receptors such as CCR4, CCR10, and CLA for the skin^32,33^. These receptors enable effector T cells to bind leukocyte adhesion molecules such as E-selectin, ICAM-1, and VCAM-1 expressed by vascular endothelial cells, while migration into the skin is facilitated by chemokines like CCL17 and CCL27^32,33^. Consistent with this, we found that the expression of adhesion molecules and chemokines was increased in the vasculature and skin, respectively, of lymphedematous tissues^34,35^. This suggests that skin homing to lymphedematous tissue is actively regulated and may represent a therapeutic target.

Consistent with prior studies of chronic inflammation in lymphedema^4,7^, the adoptively transferred CD4^+^ cells differentiated into both Th1 and Th2 cells and to a greater degree in mice that endured lymphatic injury compared to those that had not (sham surgery) (Fig. 2). Looking more carefully, a greater proportion underwent Th2 rather than Th1 differentiation, which is consistent with reports that Th2 cells in particular drive the development of secondary lymphedema^4,7^. Further evaluation is required to further clarify the interplay between Th1 and Th2 cells, in addition to the roles of other, rarer, CD4^+^ effector cells, but such results corroborate the potential benefit of targeting Th2 cells in lymphedema.

Lymphedema is characterized by edema, fibroadipose deposition, impaired lymphangiogenesis, and dysfunctional lymphatic vessels. In this study, we found that CD4^+^ T cells directly or indirectly mediate these pathologic findings. Adoptive transfer of CD4^+^ T cells with subsequent differentiation into Th2 cells corresponded with a marked increase in soft tissue fibrosis following lymphatic injury much like that seen in other fibroproliferative disorders, which are known to be driven by profibrotic Th2-derived cytokines such as IL-4, IL-13, and TGF-β1^36,37^. The accumulation of CD4^+^ T cells also negatively affected lymphangiogenesis, a finding that is consistent with studies demonstrating the effect of these cells on lymph node lymphangiogenesis in response to various stimuli^38^. Knowing that regulation of lymphangiogenesis in lymphedema is regulated by a balance between pro- and anti-lymphangiogenic forces^4–6^, it is likely that chronic accumulation of CD4^+^ cells impairs lymphatic function by actively preventing development of functional collateral vessels, thereby acting in a feed-forward manner to promote pathologic changes.

A key finding in our study was that iNOS expression is a major regulator of impaired lymphatic vessel pumping. In contrast to CD4KO mice that developed inflammation with perilymphatic iNOS^+^ inflammatory cells and impaired lymphatic transport following adoptive transfer of CD4^+^ T cells after lymphatic injury, similarly injured transgenic mice lacking iNOS had relatively normal lymphatic pumping. Similarly, studies have shown that disruption of endogenous NO gradients by inflammatory cells decreases lymphatic pumping in other inflammatory skin conditions^39,40^. Furthermore, our results suggest CD4^+^ T cells indirectly contribute by stimulating macrophages, which are potent producers of iNOS^41^, as both AT and WT mice had increased perilymphatic macrophage accumulation after lymphatic injury. This was corroborated by in vitro studies showing that treatment of macrophages with Th1 and Th2 cytokines results in increased iNOS expression (Supplementary Fig. 8). Impaired pumping may also be related to CD4^+^ T cell-dependent proliferation of α-SMA around collecting lymphatic vessels with resultant luminal obstruction and fibrosis. This effect is likely a response to increased afterload due to lymphatic vessel ligation and impaired collateral vessel formation^42,43^, a phenomenon supported by studies demonstrating a relationship between elevated afterload in patients with breast cancer-related lymphedema and diminished lymphatic pumping^44,45^. Although further research is necessary to further clarify these mechanisms, taken together, our data indicate that CD4^+^ T cells contribute to lymphatic function regulation through a variety of means, such as iNOS production and fibrosis.

Ultimately, the elucidation of the mechanisms by which CD4^+^ T cells promote lymphedema is important because it allows for the identification of potential therapeutic targets. The majority of research for treatment has focused on increasing lymphangiogenesis to bypass damaged vessels^46,47^, but this approach is limited because lymphangiogenic factors contribute to tumor growth and metastasis^48^. On the basis of our proposed sequence of events (Fig. 7), it may be possible to prevent lymphedema by inhibiting CD4^+^ T cell migration into lymph nodes, activation of CD4^+^ T cells in lymph nodes, emigration of activated CD4^+^ T cells out of lymph nodes, the adhesion molecules that guide translocation of CD4^+^ T cells from the circulation, the chemokine gradients that mediate CD4^+^ T cell skin-homing, or the effects of CD4^+^ T cells already in the skin. In this study, we chose evaluate the feasibility of blocking CD4^+^ T cell emigration from lymph nodes because FTY720, also known as fingolimod or Gilenya®, is already an FDA-approved treatment for relapsing-remitting multiple sclerosis and chronic inflammatory demyelinating polyneuropathy that has been well-characterized (Fig. 5)^49,50^. FTY720 reduces peripheral lymphocyte counts by blocking S1P receptors, which are involved in immune cell trafficking, among other roles^49^. Of note, FTY720 synergizes with calcineurin inhibitors, which is important because we have previously shown that lymphedema can be ameliorated using a topical form of the calcineurin inhibitor tacrolimus^49,51^. We found that FTY720 alone is effective in treating mouse models of lymphedema (Fig. 8), but additional research is necessary to determine its effect in combination with other agents such as tacrolimus. Given that the current treatment of lymphedema consists primarily of compression and massage therapy, pharmaceutical therapies comprise a key unmet need, so the findings of such treatment studies have the potential of changing the standard of care for this morbid disease.

In summary, we have provided insight into the spatiotemporal patterns and effects of CD4^+^ T cells, which are known to have a critical role in the development of lymphedema^4,6,27,52^. This allowed us to determine cellular pathways that may be targeted by pharmaceutical agents. In particular, this study has provided preliminary evidence for the use of FTY720, an inhibitor of T cell release from lymph nodes, as a potential treatment option, although future studies are necessary for effective clinical translation.

## Methods

### Mouse models

Experimental protocols were reviewed and approved by the Institutional Animal Care and Use Committee at Memorial Sloan Kettering Cancer Center, which adheres to the National Institutes of Health (NIH) Guide for Care and Use of Laboratory Animals and operates under the Animal Welfare Act and Animal Scientific Procedures Act of 1986. Adult female C57BL/6J (WT), CD4KO (B6.129S2-*Cd4tm1Mak*/J), CD4-eGFP (B6.NOD-Tg[Cd4-EGFP]1Lt/J), Pep Boy (B6.SJL-*Ptprca Pepcb*/BoyJ), CD28KO (B6.129S2-*Cd28tm1Mak*/J), iNOS KO (B6.129P2-*Nos2tm1Lau*/J) (all from The Jackson Laboratory; Bar Harbor, Maine), and RAG2/OTII (B6.129S6-*Rag2*^*tm1Fwa*^ Tg[TcraTcrb]425Cbn; Taconic Biosciences; Rensselaer, New York) mice utilized in this study were aged 10-14 weeks and maintained in a pathogen-free, temperature- and light-controlled environment. Animals were randomly assigned to the treatment or control group in relevant experiments and all experiments had a minimum of four animals.

We used both the tail surgery and PLND mouse models of lymphedema as these models have different strengths and weaknesses^10^. In the tail surgery model, a 3–5 mm portion of the mid-tail skin (2 cm from the base) is circumferentially excised and the deep lymphatic collecting vessels are ligated after identification using Evans’ blue, which is injected into the distal tail. This reliably results in tail swelling, severe impairment in lymphatic function, and histopathologic features consistent with clinical lymphedema for as long as 10 weeks post-operatively^5,53–55^. In the PLND model, an incision is made in the area of the popliteal lymph node, which is identified after an injection of Evans’ blue into the dorsal hindfoot and removed with its surrounding fat pad to include the efferent and afferent lymphatic vessels; the incision is then closed with 3-0 non-absorbable sutures in a continuous fashion. Although sustained edema is not observed after PLND, this model is useful for evaluation of collateral lymphatic vessel formation and collecting lymphatic vessel pumping^56^. Mice that underwent sham surgery (skin incision without lymphatic vessel ligation or lymphadenectomy) were used as controls when necessary. Animals were excluded and killed if wound infection or skin ulceration was observed.

During all surgical procedures, anesthesia was induced with isoflurane (Henry Schein Animal Health; Dublin, OH). Respiratory rate and tail pinching were used to monitor the depth of anesthesia. Tissue harvest was performed after animals were killed by carbon dioxide asphyxiation as recommended by the American Veterinary Medical Association.

### NK1.1 depletion

To determine whether NK1.1 depletion would affect the lymphedema phenotype, we intraperitoneally injected 100 μL of 200 μg of monoclonal antibodies to NK1.1 (PK136; #BE0036; Bio X Cell; West Lebanon, NH) or isotype control diluted in phosphate-buffered saline (PBS; Thermo Fisher Scientific; Waltham, MA) into WT mice after tail skin and lymphatic excision (Supplementary Fig. 2a). These dosages were determined based on a modification of previously published protocols^57,58^. Injections were started 2 weeks post-operatively and were performed every 4 days for a total of 4 weeks. Mice were killed for analysis 2 days after the last injection.

### Adoptive transfer

Adoptive transfer of naive CD4^+^ T cells to CD4KO mice was performed after tail skin and lymphatic excision (Fig. 1a) or PLND (Fig. 1f). CD4^+^ T cells were isolated from the spleens of WT, CD4-eGFP, or RAG2/OTII mice by negative selection using magnetic beads per the manufacturer’s recommendations (#130-104-453, Miltenyi Biotech; Auburn, CA). Flow cytometric analysis on single-cell suspensions was used to confirm majority of isolated CD4^+^ T cells were naive (CD44^−^CD62L^+^) (Supplementary Fig. 2a). In one experiment, we also followed the manufacturer’s recommendations for two PKH fluorescent cell linker kits (MINI26 and MINI67; Sigma-Aldrich; St. Louis, MO) to label WT mouse-derived CD4^+^ T cells to allow for differential tracking of cells adoptively transferred to CD4KO mice 24 and 48 h before harvest. Approximately 1 × 10^7^ CD4^+^ T cells suspended in 100 μL of PBS were adoptively transferred via retroorbital injection starting 2 weeks after surgery. Successful transfer was confirmed by flow cytometric analysis of splenic cells (Supplementary Fig. 3b). Control CD4KO mice that did not undergo adoptive transfer were injected with equivalent amounts of PBS alone.

We also performed adoptive transfer with DCs harvested from female Pep Boy mice, which possess the differential pan-leukocyte marker CD45.1, to allow for tracking of DCs following PLND. CD11c^+^ DCs were similarly isolated using magnetic beads per the manufacturer’s recommendations (#130-052-001; Miltenyi Biotech). Approximately 2 × 10^7^ CD45.1^+^CD11c^+^ DCs were suspended in 100 μL of PBS and retroorbitally injected into CD4KO or WT mice 2 weeks after PLND or sham surgery (Fig. 5a).

### Tail volume measurements and analysis of lymphatic function

Tail volumes (*V*) following tail skin and lymphatic excision were calculated weekly using the truncated cone formula:

1 $$V = 1/4\pi \,\left( {C_1C_2 + C_2C_3 + C_3C_4} \right),$$

in which *C* represents serial circumference measurements obtained using digital calipers every 1 cm starting at the surgical site going distally toward the tip of the tail.

Lymphoscintigraphy was used to assess lymphatic transport function in the tail^5,53^. In this technique, 50 μL of ^99m^Tc (Nuclear Diagnostic Products; Rockaway, NJ) was injected into the distal tails of appropriate mice. Images were then obtained using an X-SPECT camera (Gamma Medica; Northridge, CA) and region-of-interest (ROI) analysis was performed using ASIPro Software (CTI Molecular Imaging; Knoxville, TN) to calculate peak and rate of decay-adjusted nodal uptake in the sacral lymph nodes.

Lymphoscintigraphy findings were correlated with evaluation of ipsilateral hindlimb collecting lymphatic vessel pumping following PLND^59,60^. After anesthesia was induced, 15 μL of 0.15 mg/mL of ICG (Sigma-Aldrich) diluted in sterile water was intradermally injected into the first webspace of the dorsal hindlimbs. Mice were then allowed to awaken and move freely for 30 min prior to being placed under anesthesia again for near-infrared imaging of the hindlimb using an EVOS EMCCD camera (Life Technologies; Carlsbad, CA) and a LED light source (CoolLED; Andover, UK) mounted on a Zeiss SteREO Lumar V12 microscope (Zeiss; Jena, Germany). A ROI over the dominant collecting vessel was identified and lymphatic pumping function was analyzed using Fiji software (NIH; Bethesda, MD) to subtract the background fluorescent intensity plotted over time.

### FTY720 treatment

To test our hypothesis that FTY720 treatment would prevent the development of lymphedema, we randomized WT or AT mice to treatment with FTY720 (Sigma-Aldrich) or vehicle control. FTY720 was dissolved in water per the manufacturer’s recommendations and supplied ad libitum starting post-operative day zero (Fig. 8a, d). Mice that underwent PLND were treated for 3 weeks, whereas those mice that underwent tail skin and lymphatic excision were treated for 6 weeks before analysis. All mice received ~1.25 mg/kg/day and the water was changed every 3 days.

### Histology and immunohistochemistry

Harvested tissues were fixed in 4% paraformaldehyde (Affymetrix, Inc.; Cleveland, OH) at 4 °C, embedded in paraffin, and sectioned at 5 µm. All sections were rehydrated prior to hematoxylin and eosin (H&E) and immunofluorescent staining.

H&E staining was performed with Mayer’s hematoxylin (Lillie’s Modification; Dako North America; Carpinteria, CA) and eosin Y solution (Thermo Fisher Scientific; Waltham, MA). After alcohol-based dehydration and alcohol extraction with xylene, sections were mounted using VectaMount Permanent Mounting Medium (Vector Laboratories, Inc.; Burlingame, CA).

For immunofluorescent staining, heat-mediated antigen unmasking was achieved using sodium citrate (Sigma-Aldrich) in a 90°C water bath. Non-specific binding was blocked with 20% donkey serum (Sigma-Aldrich) and 80% PBS for 1 h in room temperature. Sections were then incubated at 4 °C with the appropriate primary antibody overnight. The following primary antibodies were used: rat monoclonal anti-CD45 (1:100; 30-F11; #MAB114), rat monoclonal anti-CD4 (1:100; GK1.5; #BAM554), goat polyclonal anti-podoplanin (1:100; #AF3244), and goat polyclonal anti-LYVE-1 (1:400; #2125-LY) from R&D Systems (Minneapolis, MN); rabbit polyclonal anti-collagen I (1:100; #ab34710), rat monoclonal anti-IL-4 (1:100; #11524), rabbit polyclonal anti-IFN-γ (1:100; #ab9657), rabbit polyclonal anti-CCL17 (1:100; #ab182793), rat monoclonal anti-CCL27 (1:100; 6M35; #ab194649), rabbit polyclonal anti-E-selectin (1:100; #ab18981), rabbit monoclonal anti-VCAM-1 (1:500; EPR5047; #ab134047), rat monoclonal anti-ICAM-1 (1:100; YN1/1.7.4; #ab119871), and rabbit polyclonal anti-iNOS (1:100; #ab3523) from Abcam (Cambridge, MA); and mouse monoclonal anti-α-SMA (1:100; 1A4; #A2547) from Sigma-Aldrich. After washing, sections were incubated with corresponding fluorescent-labeled secondary antibody conjugates (1:1000; Life Technologies) for 5 h and 4,6-diamidino-2-phenylindole (DAPI; #D1306; Molecular Probes/Invitrogen; Eugene, OR) for 10 minutes before mounting with Mowiol (Sigma-Aldrich).

All sections were scanned using a Mirax slide scanner (Zeiss) and evaluated with brightfield for H&E or fluorescent microscopy for immunofluorescent staining. Analysis was completed using Pannoramic Viewer (3D Histech; Budapest, Hungary). Cell counts and calculation of capillary lymphatic vessel area were performed on high-powered sections (40–80×) with 4–5 high-powered fields (hpf) per animal by two blinded reviewers. To quantify perilymphatic cells, ImageJ software (NIH; available at https://imagej.nih.gov) was used to identify the positive cells within 50 µm of LYVE-1^+^ lymphatic vessels. Type I collagen deposition was quantified as a ratio of positively stained dermis and subcutaneous tissues within a fixed threshold to total tissue area using Metamorph Offline Software (Molecular Devices; Sunnyvale, CA)^61^. Subcutaneous fibroadipose thickness was analyzed as the width of tissues bounded by the reticular dermis to the deep fascia in four standardized regions of tail cross-sections.

### Flow cytometry

Flow cytometry was performed using single-cell suspensions obtained from skin, lymph nodes, spleen, and bone marrow. For tail and hindlimb skin, the tissues were harvested 1 cm distal to the surgical site. Single-cell suspensions were created by mechanical dissociation followed by enzymatic digestion with DNase I, Dispase II, collagenase D, and collagenase IV (Roche Diagnostics; Indianapolis, IN). Erythrocytes in the bone marrow, spleen, and lymph nodes were lysed with RBC lysis buffer (eBioscience; San Diego, CA). All suspensions were filtered through 100 and 40 μm filters. Non-specific staining was reduced by using Fc receptor block (rat monoclonal anti-CD16/CD32; #14-0161-85; eBioscience). Cells were then stained with varying combinations of the following fluorophore-conjugated monoclonal antibodies: mouse anti-CD45.1 (A20; #11-0453-081), rat anti-CD3 (17A2; #17-0032-80), rat anti-CD4 (GK1.5; #11-0041-82, 12-0041-81), Armenian hamster anti-CXCR3 (CXCR3-173; #45-1831-80), Armenian hamster anti-CCR5 (7A4; #12-1951-82), and rat anti-MHCII (M5/114.15.2; #17-5321-82) from eBioscience; and rat anti-CD45 (30-F11; #103139), rat anti-CD4 (GK1.5; #100429), rat anti-CD44 (IM7; #103011), rat anti-CD62L (MEL-14; #104408), and rat anti-CD86 (CL-1; #105007) from BioLegend (San Diego, CA); and rat anti-161/NK1.1 (694370; #FAB7614G) from R&D Systems (1:150 for all).

Zombie NIR^TM^ (#423105, BioLegend) or DAPI viability stains were also used on all samples to allow for exclusion of dead cells. Single-stain compensation samples were created using UltraComp eBeads^TM^ (#01-2222-42, Affymetrix, Inc.; San Diego, CA). Flow cytometry was performed using a BD Fortessa flow cytometry analyzer (BD Biosciences; San Jose, CA) with BD FACS Diva and data were analyzed with FlowJo software (Tree Star; Ashland, OR). Gating strategies are shown in Supplementary Fig. 1.

### Macrophage isolation and treatment

To assess the effect of T cell-derived cytokines on macrophage differentiation, monocytes were harvested from bone marrow of WT mice^62^. Briefly, cells were flushed from the femurs and tibias of mice using a 30-gauge needle, filtered through a 100 μm filter, and treated with RBC lysis buffer (eBioscience). Cells were then plated onto sterile petri dishes and cultured in Roswell Park Memorial Institute (RPMI) 1640 (Gibco; Carlsbad, CA) with 20% fetal calf serum (Gibco), 2.4% penicillin-streptomycin (Gibco), and 1× GlutaMAX (Invitrogen) plus 20 ng/mL murine recombinant M-CSF (R&D Systems). Media change was performed every 2–3 days. After 1 week, cells were left untreated or treated with recombinant IFN-γ (10 ng/mL; Peprotech; Rocky Hills, NJ), IL-4 (20 ng/mL; Peprotech), or IFN-γ and IL-4 combined for 48 h before analysis.

### Quantitative PCR analysis

PCR analysis for iNOS (Mm_Nos2_1_SG, #QT00100275; Qiagen; Hilden, Germany) and Arginase-1 (Mm_Arg1_1_SG, #QT00134288; Qiagen) expression was performed on the bone marrow-derived macrophages. RNA isolation was achieved using RNeasy Mini Kit (Qiagen) per the manufacturer’s recommendations. cDNA synthesis was carried out using Maxima H-minus Reverse Transcriptase (Invitrogen). Real-time PCR was performed on a Viia7 PCR system (Invitrogen) with Qiagen Quantitect SYBR Green reagent at 55 °C for annealing temperature and 40 cycles of amplification. All samples were assessed in duplicate.

### Statistical analysis

Statistical analysis was performed using GraphPad Prism (GraphPad Software, Inc., San Diego, CA). Student’s *t*-test was used to compare differences between two groups, while comparisons between three or more groups were conducted using one- or two-way analysis of variance (ANOVA) with the Brown–Forsythe test for equality of group variances and the Tukey’s multiple comparisons test for individual group comparisons. Data are presented as mean ± standard deviation (s.d.) unless otherwise noted and *P* < 0.05 was considered significant.

### Data availability

The data that support the findings of this study are available from the corresponding author upon request.

## Electronic supplementary material

Supplementary Information

Description of Additional Supplementary Files

Supplementary Movie 1

Supplementary Movie 2

Supplementary Movie 3

Supplementary Movie 4
