# Supplementary material for: CD4+ T cells are activated in regional lymph nodes and migrate to skin to initiate lymphedema
Source: Nat Commun. 2018 May 17;9:1970. doi: 10.1038/s41467-018-04418-y (PMC5958132; doi:10.1038/s41467-018-04418-y)
Supplement: Supplementary file 1 — Supplementary Information [file 41467_2018_4418_MOESM1_ESM.pdf]

Supplementary Figures

$CD4^+$ T cells are activated in regional lymph nodes and home to skin to initiate lymphedema

García Nores et al.

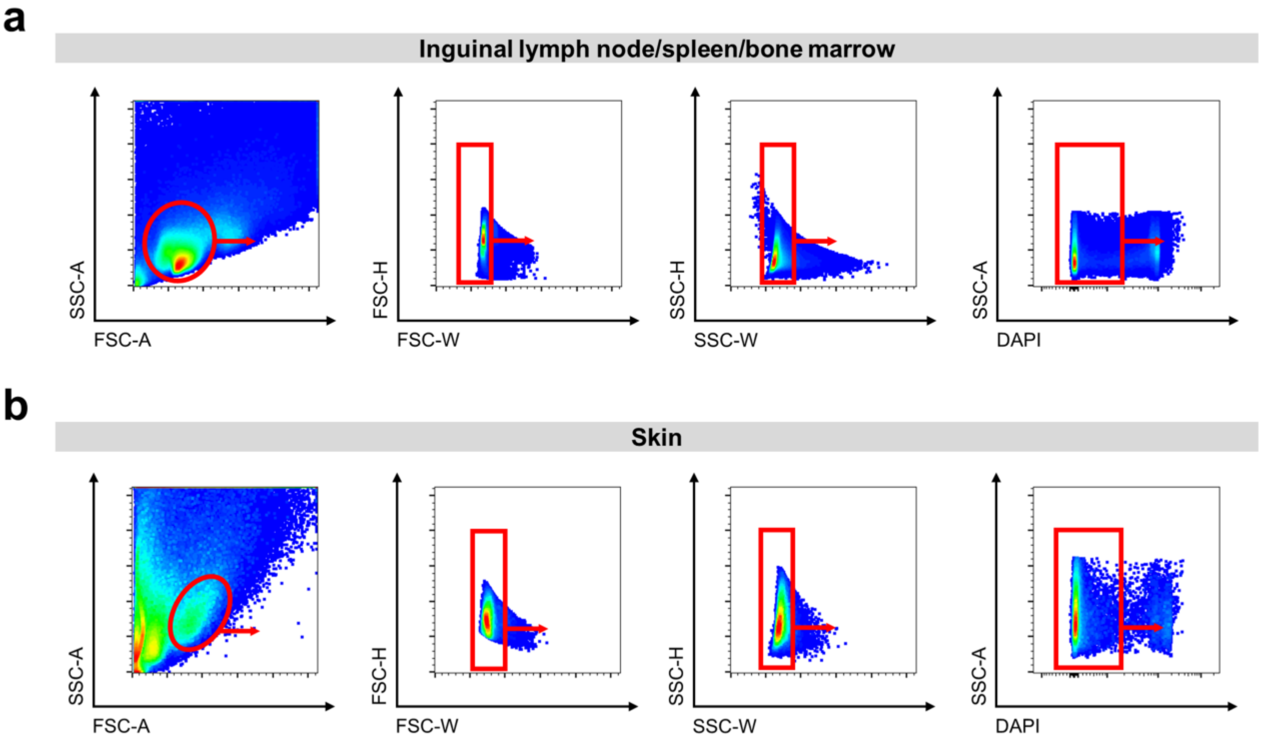

Supplementary Fig. 1. Gating strategy for flow cytometry. a, b CD4^{+} T cells and subsets were identified in single-cell suspensions obtained from ipsilateral inguinal lymph nodes, spleen, bone marrow (**a**), and skin from the tail or hindlimb distal to the surgical site (**b**) following tail skin and lymphatic excision or popliteal lymph node dissection, respectively, using a standard gating strategy. This gating strategy was utilized for Fig. 2b, 2d, 2f, 3b-c, 3e, 8d-e; Supplementary Fig. 1f, 2a-b, 3d-e, and 7.

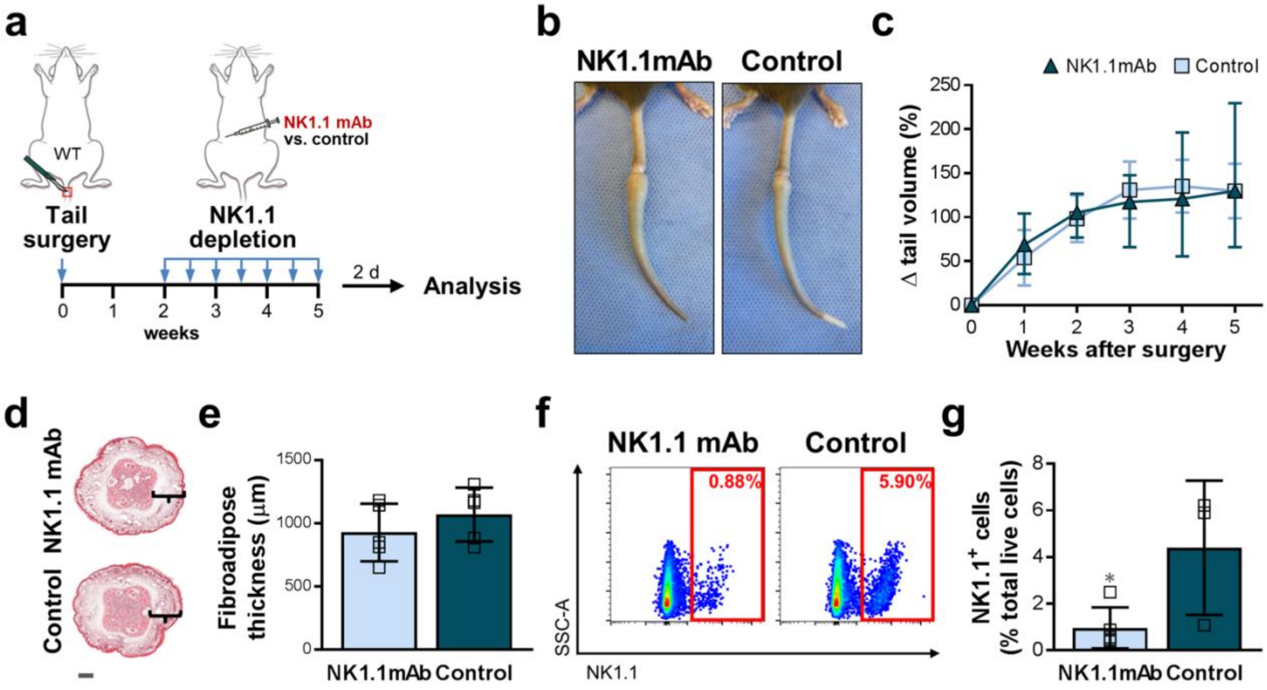

Supplementary Fig. 2. NK1.1 depletion does not reverse lymphedema. a Schematic diagram of treatment with neutralizing antibodies to NK1.1 or isotype control following tail skin and lymphatic excision. Mice sacrificed 37 days after surgery. **b** Representative photographs of tails five weeks after surgery. **c** Quantification of tail volume change (n=5/group). **d** Representative H&E staining of tail cross-sections with brackets indicating fibroadipose tissue; scale bar, 1000 μm. **e** Quantification of fibroadipose thickness (n=5/group; 5 hpf/mouse). **f** Representative FACS plots (*left*) and quantification (*right*) of single, live NK1.1^{+} cells in tail skin (n=3 for control group, n=6 for NK1.1 mAb group). Data representative of a minimum of 2 independent experiments with similar results; statistical analyses of one experiment shown. Mean ± s.d.; *P<0.05 by two-way ANOVA or unpaired student's t test. H&E, hematoxylin and eosin; hpf, high-powered field.

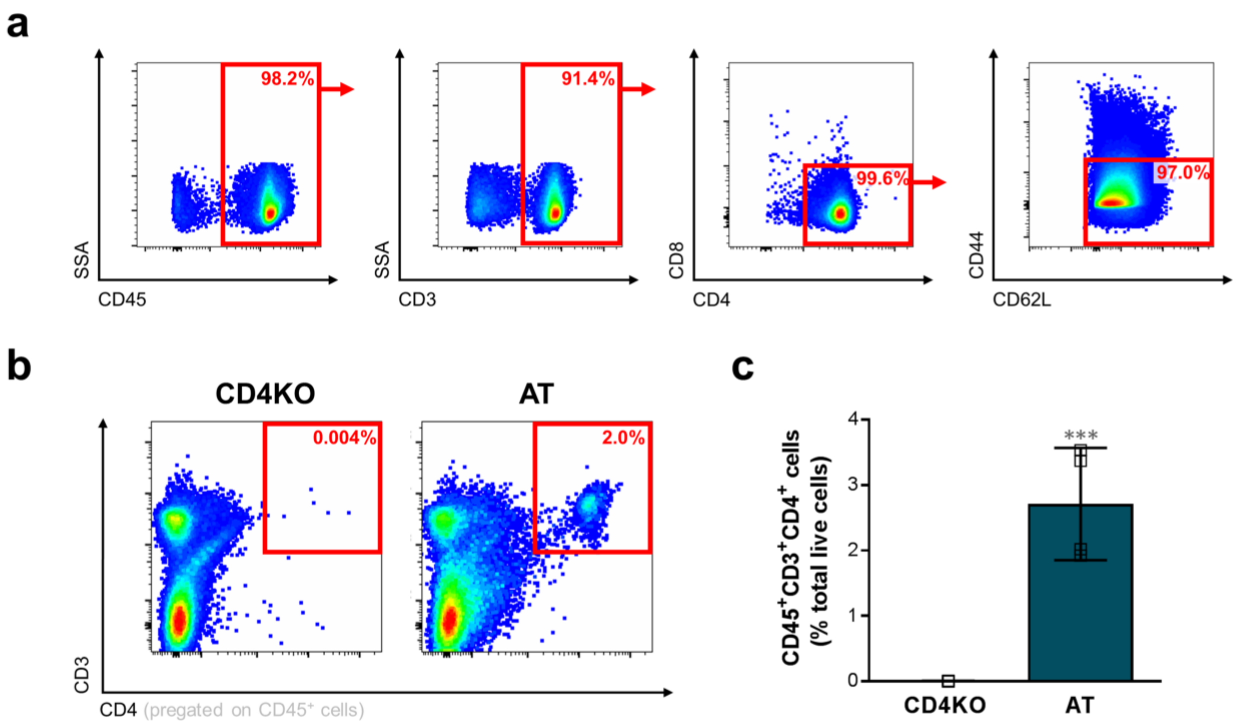

Supplementary Fig. 3. Naïve CD4$^+$ T cells were successfully isolated and transferred to CD4KO mice. a Representative FACS plots depicting the gating strategy utilized to identify single, live CD45$^+$CD3$^+$CD4$^+$CD44$^-$CD62L$^+$ cells in single-cell suspensions of cells isolated from WT mouse spleens by magnetic bead negative selection. **b, c** Representative FACS plots (**b**) and quantification (**b**) of single, live CD45$^+$CD3$^+$CD4$^+$ cells in the spleens of CD4KO and AT mice (n=4 for AT group, n=5 for CD4KO group; mean ± s.d.; ***P=0.0002 by unpaired student's t test). Data representative of a minimum of 2 independent experiments with similar results; statistical analyses of one experiment shown. AT, CD4KO mice that underwent adoptive transfer with naïve CD4$^+$ T cells.

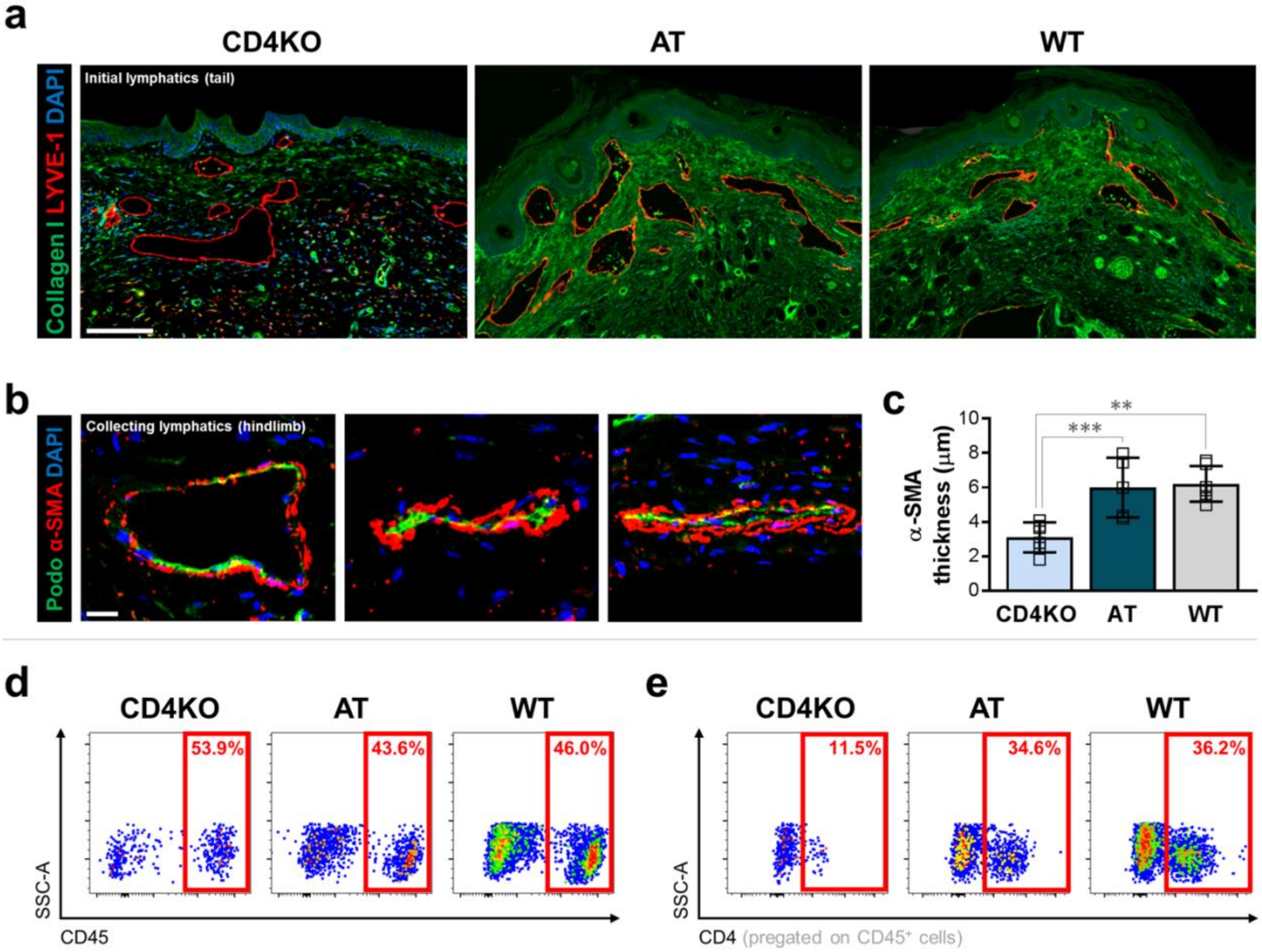

Supplementary Fig. 4. Adoptive transfer of $CD4^+$ T cells to CD4KO mice results in increased fibrosis and local inflammation in area of lymphatic injury. Mice sacrificed 6 weeks after tail skin and lymphatic excision or 4 weeks after PLND. **a** Representative immunofluorescent images of tail cross-sections co-localizing LYVE-1^+ initial lymphatic vessels with type I collagen; scale bar, 200 µm. **b** Representative immunofluorescent images of hindlimb collecting lymphatic vessels co-localizing podoplanin and α-SMA; scale bar, 100 µm. **c** Quantification of α-SMA thickness (n=6/group; 4 hpf/mouse; mean ± s.d.; ***P*<0.01 and ****P*<0.001 by one-way ANOVA with Tukey's multiple comparisons test). **d, e** Representative FACS plots of single, live $CD45^+$ (**d**) and $CD45^+CD4^+$ cells (**e**) in tail skin. Data representative of a minimum of 2 independent experiments with similar results; statistical analyses of one experiment shown. α-SMA, alpha smooth muscle actin; AT, CD4KO mice that underwent adoptive transfer with naïve $CD4^+$ T cells; hpf, high-powered field; PLND, popliteal lymph node dissection.

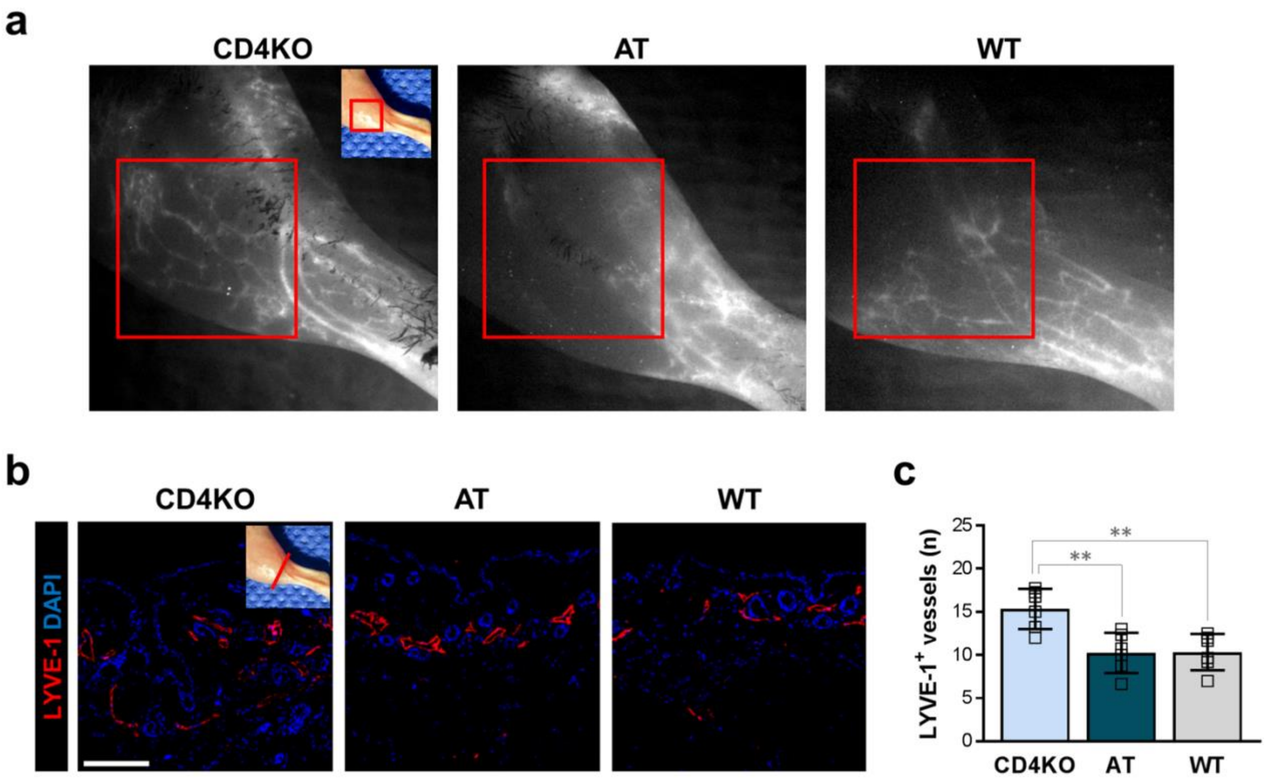

Supplementary Fig. 5. Adoptive transfer of CD4$^+$ T cells to CD4KO mice results in decreased collateral vessel formation. Mice analyzed 4 weeks after PLND. **a** Representative lymphangiography images after ICG injection with red squares indicating the presence of collateral vessel formation in CD4KO mice and the absence in AT and WT mice. **b** Representative immunofluorescent images localizing LYVE-1$^+$ initial lymphatic vessels with inset for anatomic correlation; scale bar, 200 µm. **c** Quantification of LYVE-1$^+$ vessels per 0.25 mm^2 (n=6/group; 4 hpf/mouse; mean ± s.d.; ***P*<0.01 by one-way ANOVA with Tukey's multiple comparisons test). Data representative of a minimum of 2 independent experiments with similar results; statistical analyses of one experiment shown. AT, CD4KO mice that underwent adoptive transfer with naïve CD4$^+$ T cells; hpf; high-powered field; ICG, indocyanine green.

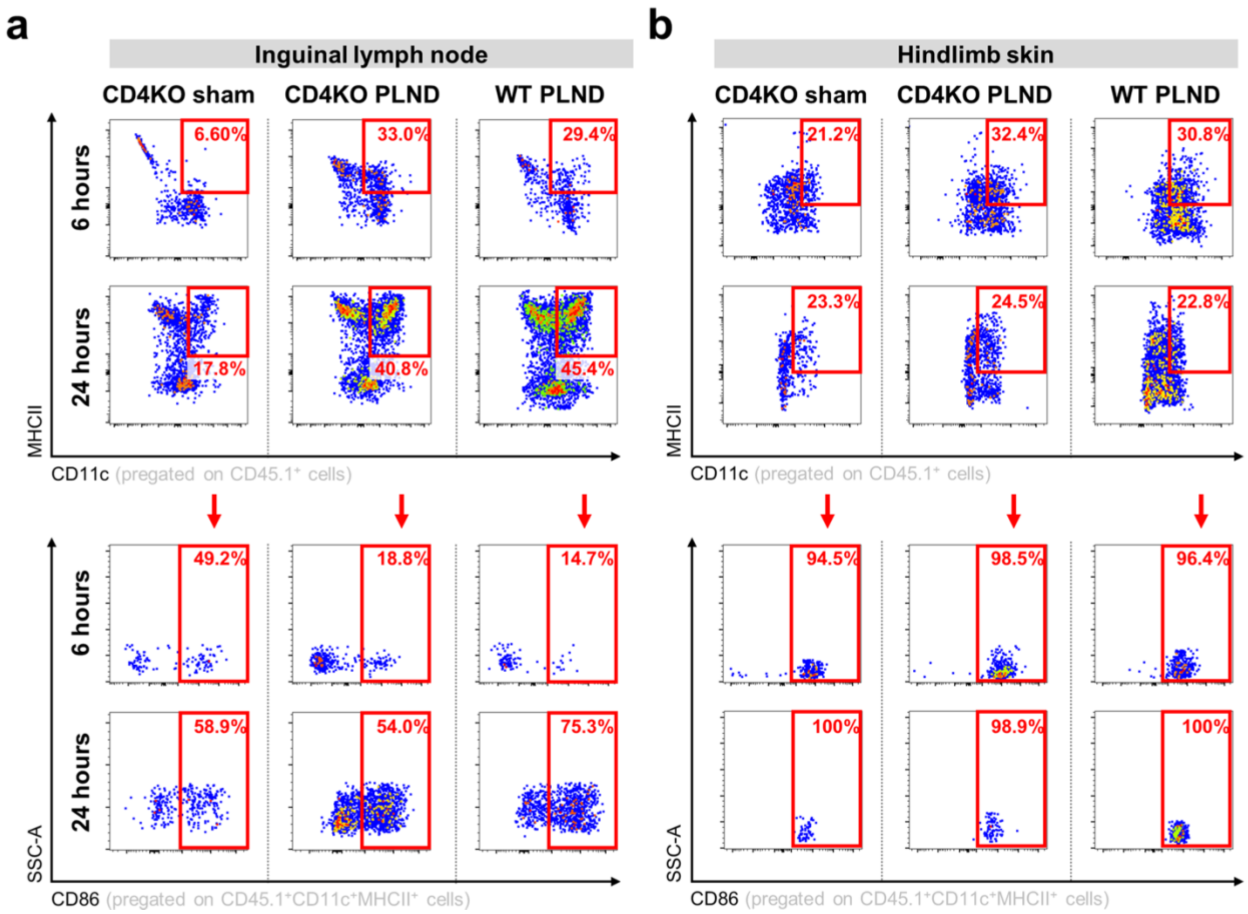

Supplementary Fig. 6. Adoptively transferred DCs are initially activated in the skin after lymphatic injury. a, b Representative FACS plots of single, live CD45.1$^+$CD11c$^+$MHCII$^+$ DCs (*upper*) further characterized into activated CD45.1$^+$CD11c$^+$MHCII$^+$CD86$^+$ DCs (*lower*) in the ipsilateral inguinal lymph nodes (**a**) and hindlimb skin (**b**) of mice depicted in Fig. 5a. DCs, dendritic cells; PLND, popliteal lymph node dissection.

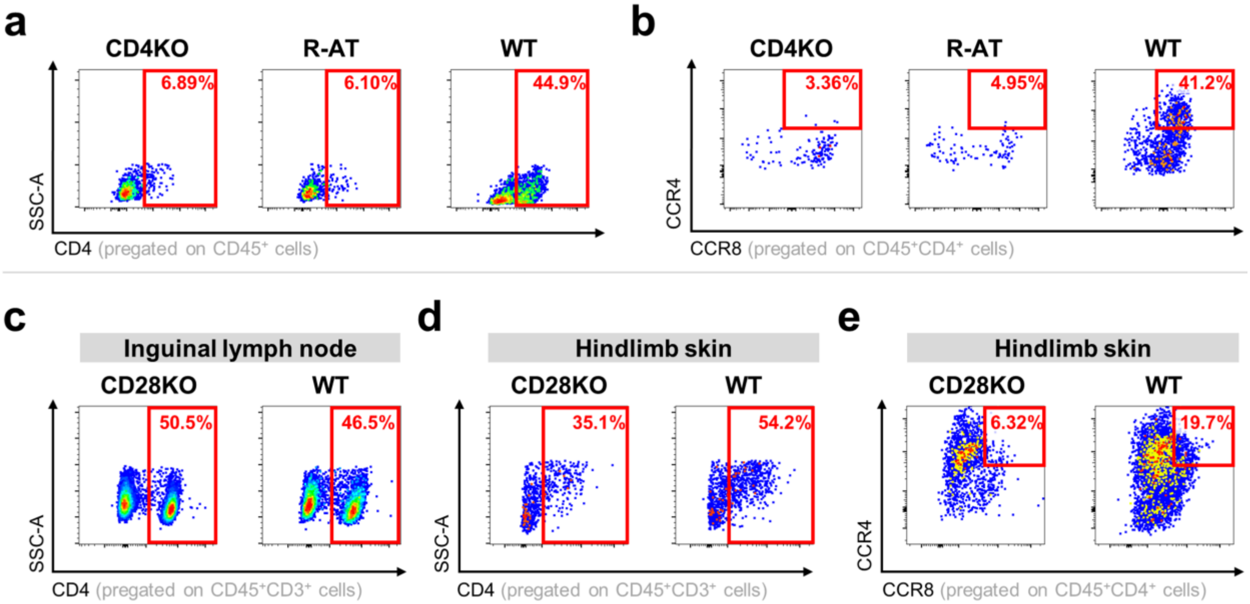

Supplementary Fig. 7. T cell activation is required for the development of lymphedema. a, b Representative FACS plots of single, live $CD45^+CD4^+$ cells (**a**) and $CD45^+CD4^+CCR4^+CCR8^+$ Th2 cells (**b**) in hindlimb skin of mice from experiment depicted in Fig. 5c. **c, d** Representative FACS plots of single, live $CD45^+CD3^+CD4^+$ cells in ipsilateral inguinal lymph nodes (**c**) and hindlimb skin (**d**) of mice from experiment depicted in Fig. 5f. **e** Representative FACS plots of single, live $CD4^+CCR4^+CCR8^+$ Th2 cells in hindlimb skin of mice from experiment depicted in Fig. 5f.

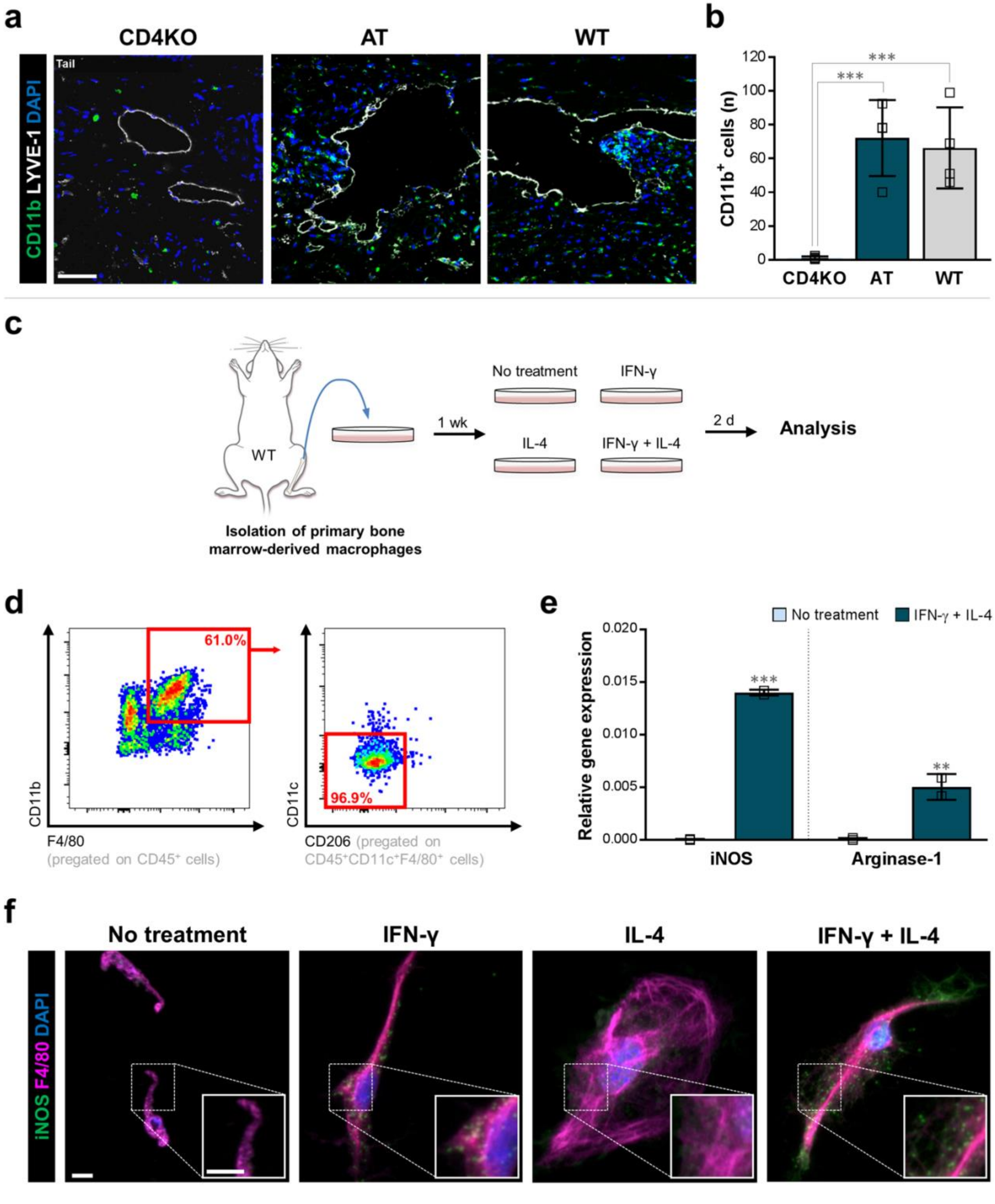

Supplementary Fig. 8. T cell cytokines promote the accumulation of iNOS-producing macrophages. a Representative immunofluorescent images of tail cross-sections co-localizing LYVE-1$^+$ initial lymphatic vessels with CD11b 6 weeks after tail skin and lymphatic excision; scale bar, 50 μm. **b** Quantification of CD11b$^+$ macrophages within 50 μm of LYVE-1$^+$ lymphatic vessels (n=4 for WT, n=5 for CD4KO and WT groups; 4 hpf/mouse). **c** Schematic diagram of macrophage isolation and subsequent *in vitro* treatment. **d** Representative FACS plots of single, live cultured CD45$^+$CD11b$^+$F4/80$^+$CD11c$^-$CD206$^-$ macrophages following isolation and prior to treatment. **e** PCR quantification of iNOS (*left*) and Arginase-1 (*right*) expression in cultured cells (n=2 for combined treatment, n=3 for no treatment, samples run in duplicate). **f** Representative immunofluorescent images co-localizing iNOS and F4/80 in cultured cells with insets representing magnified views; scale bars, 5 μm. Mean ± s.d.; **P*<0.05, ***P*<0.01, and ****P*<0.001 by one-way ANOVA with Tukey's multiple comparisons test or student's t test. AT, CD4KO mice that underwent adoptive transfer with naïve CD4$^+$ T cells.
