# Supplementary material for: CD4+ T cells are activated in regional lymph nodes and migrate to skin to initiate lymphedema
Source: Nat Commun. 2018 May 17;9:1970. doi: 10.1038/s41467-018-04418-y (PMC5958132; doi:10.1038/s41467-018-04418-y)
Supplement: Supplementary file 2 — Description of Additional Supplementary Files [file 41467_2018_4418_MOESM2_ESM.pdf]

Description of Additional Supplementary Files

Supplementary Movie 1. No impairment lymphatic vessel pumping despite lymphatic injury in CD4KO mice. Near-infrared lymphangiography of collecting lymphatic vessel pumping following PLND in CD4KO mice.

Supplementary Movie 2. Adoptively transferred $CD4^+$ T cells mediate impaired lymphatic vessel pumping. Near-infrared lymphangiography of collecting lymphatic vessel pumping following PLND in AT mice.

Supplementary Movie 3. Impaired lymphatic vessel pumping after lymphatic injury in WT mice. Near-infrared lymphangiography of collecting lymphatic vessel pumping following PLND in WT mice.

Supplementary Movie 4. iNOS mediates impaired lymphatic vessel pumping following lymphatic injury. Near-infrared lymphangiography of collecting lymphatic vessel pumping following PLND in iNOS knockout (iNOS KO) mice.
